# Like Will to Like: Abundances of Closely Related Species Can Predict Susceptibility to Intestinal Colonization by Pathogenic and Commensal Bacteria

**DOI:** 10.1371/journal.ppat.1000711

**Published:** 2010-01-08

**Authors:** Bärbel Stecher, Samuel Chaffron, Rina Käppeli, Siegfried Hapfelmeier, Susanne Freedrich, Thomas C. Weber, Jorum Kirundi, Mrutyunjay Suar, Kathy D. McCoy, Christian von Mering, Andrew J. Macpherson, Wolf-Dietrich Hardt

**Affiliations:** 1 Institute of Microbiology, ETH Zürich, Zürich, Switzerland; 2 Institute of Molecular Biology and Swiss Institute of Bioinformatics, University of Zürich, Zürich, Switzerland; 3 Gastroenterology Inselspital, Department Klinische Forschung, Bern, Switzerland; 4 Faculty of Health Sciences, McMaster University, Hamilton, Ontario, Canada; 5 School of Biotechnology, KIIT University, Bhubaneswar, Orissa, India; University of Arizona, United States of America

## Abstract

The intestinal ecosystem is formed by a complex, yet highly characteristic microbial community. The parameters defining whether this community permits invasion of a new bacterial species are unclear. In particular, inhibition of enteropathogen infection by the gut microbiota ( = colonization resistance) is poorly understood. To analyze the mechanisms of microbiota-mediated protection from *Salmonella enterica* induced enterocolitis, we used a mouse infection model and large scale high-throughput pyrosequencing. In contrast to conventional mice (CON), mice with a gut microbiota of low complexity (LCM) were highly susceptible to *S. enterica* induced colonization and enterocolitis. Colonization resistance was partially restored in LCM-animals by co-housing with conventional mice for 21 days (LCM^con21^). 16S rRNA sequence analysis comparing LCM, LCM^con21^ and CON gut microbiota revealed that gut microbiota complexity increased upon conventionalization and correlated with increased resistance to *S. enterica* infection. Comparative microbiota analysis of mice with varying degrees of colonization resistance allowed us to identify intestinal ecosystem characteristics associated with susceptibility to *S. enterica* infection. Moreover, this system enabled us to gain further insights into the general principles of gut ecosystem invasion by non-pathogenic, commensal bacteria. Mice harboring high commensal *E. coli* densities were more susceptible to *S. enterica* induced gut inflammation. Similarly, mice with high titers of Lactobacilli were more efficiently colonized by a commensal *Lactobacillus reuteri* ^RR^ strain after oral inoculation. Upon examination of 16S rRNA sequence data from 9 CON mice we found that closely related phylotypes generally display significantly correlated abundances (co-occurrence), more so than distantly related phylotypes. Thus, in essence, the presence of closely related species can increase the chance of invasion of newly incoming species into the gut ecosystem. We provide evidence that this principle might be of general validity for invasion of bacteria in preformed gut ecosystems. This might be of relevance for human enteropathogen infections as well as therapeutic use of probiotic commensal bacteria.

## Author Summary

The commensal microbiota, populating the intestinal tract to high levels, is fundamental to human health. It exerts beneficial effects on the immune system and contributes to protection against gastrointestinal infections ( = colonization resistance) by largely unknown mechanisms. Here, we reveal characteristics of the commensal microbiota indicative for a high or low degree of colonization resistance. Using a mouse model for *Salmonella enterica* induced gut inflammation and microbiota analysis by 454 amplicon sequencing, we show that mice having different types of microbiota exhibit differential susceptibility to pathogen infection. In addition, our data lead to the description of a new concept in gut ecosystem biology: the intrusion-success of an extrinsic bacterial species into an established gut ecosystem is related to the abundance of closely related bacteria, already present in this gut ecosystem. We show that this principle applies not only to enteropathogen infection but also to inoculation with beneficial gut bacteria. Humans can display largely different degrees of susceptibility to enteric infections. Similarly, the effectiveness of probiotic therapy varies greatly from person to person. Our data might explain these differences and could be used for increasing the efficacy of probiotic therapy and for identifying patients at risk of developing enteric infections.

## Introduction

The mammalian intestine hosts a microbial community of astonishing density and complexity. This intricate association presumably required significant coevolution of the host and its microbiota. Apparently, this coevolution has been guided by positive selection for factors that result in a state of both mutual tolerance and benefit.

Microbial colonization of the intestine takes place right after birth and complexity steadily increases henceforward. The temporal and spatial assembly of the gut microbiota is apparently not guided by specific rules but eventually, after weaning, a stable microbial ecosystem is formed [1]. The adult human intestine hosts 10^13^ to 10^14^ bacteria belonging to at least 500 different species or strains [2]. Up to 9 different bacterial phyla are usually found; however, the Firmicutes and Bacteroidetes account for over 90% of all bacteria [3]. Despite its striking conservation on a higher phylogenetic level, the abundance of bacteria on species or strain level varies extensively between non-related individuals. Nevertheless, a core gut microbiome ( = sum of microbial genes) that is shared among different individuals ensures conservation of metabolic functions provided by the microbiota [4]. It is assumed that the microbial ecosystem, once it is formed, efficiently prevents invasion by foreign species. This has been extensively studied in the case of enteric pathogens and is known as ‘colonization resistance’ (CR) [5].

The gut microbiota protects its host against infection by life-threatening pathogens such as *Vibrio cholerae*, pathogenic *Escherichia coli* strains, *Shigella* spp., *Clostridium difficile* and *Salmonella* spp. [6],[7]. To date, the molecular bases of CR as well as the key bacterial species involved remain poorly defined. It is clear that if the gut microbiota is absent or disturbed (i.e. germfree status, antibiotic treatment, gut inflammation) the infection risk increases drastically [8],[9],[10],[11],[12]. CR might not only exclude pathogenic bacteria but also acts against harmless or even beneficial bacteria, such as probiotics. For example, the efficiency of probiotic therapy can differ greatly among individuals [13],[14],[15],[16]. To increase effectiveness of probiotic therapy, research aims at improving the half-life of probiotic strains in the gut [17].

In this study we set out to identify characteristics of the bacterial gut microbiota that are linked to infectivity of the human pathogen *Salmonella enterica*. Conventional mice (CON) harbouring a complex gut microbiota are highly resistant to oral *Salmonella enterica* infection and concomitant induction of gut inflammation [18]. We tested colonization resistance of mice harbouring different types of gut microbiota. On a quantitative level, we found that mice having a higher gut microbiota complexity exhibited increased protection against *Salmonella*-induced gut inflammation. In addition we found that the invasion-success of novel species into an established gut ecosystem (i.e. *Salmonella enterica*, *Lactobacillus reuteri* ^RR^) may be predetermined by the abundance of species that are closely related to the invader.

## Materials and Methods

### Animals

We generated LCM mice by colonizing germfree mice with the Altered Schaedler flora (ASF) according to the protocol published on the Taconic webpage. Mice were inoculated at eight weeks of age by intra-gastric and intra-rectal administration of 10^7^–10^8^ c.f.u. of ASF bacteria on consecutive days (www.taconic.com/library). LCM mice (C57Bl/6 background) were maintained under barrier conditions in individually ventilated cages with autoclaved chow and autoclaved, acidified water. No mice with complex gut microbiota were housed in the same room to prevent contamination with natural gut bacteria. CON C57Bl/6 mice were obtained from Janvier (France), Charles River Laboratories (Sulzfeld, Germany), from the Rodent Center HCI (RCHCI Zürich) and the Biologisches Zentrallabor (BZL; Univeristy Hospital Zurich). CON transgene negative B6.129P-*CX3CR1^tm1Litt^*/J mice (CX3CR1) [19] and CON Ly5.1 (B6.SJL-*Ptprc^a^ Pepc^b^*) were bred at the RCHCI Zürich and CON heterozygous MyD88^+/−^ mice (C57BL/6 background) [20] at RCC Füllinsdorf, respectively. All mice were bred and kept specified pathogen free in individually ventilated cages. This restricts microbial transfers between mice housed in the same room and animal facility.

LCM mice, CON mice or streptomycin-pretreated CON mice (20 mg/animal 24h prior to *Salmonella* infection) were infected by gavage with 5×10^7^ CFU *S*. Typhimurium SL1344 wildtype or avirulent (*sseD*::*aphT* [21]) strains or *S.* Enteritidis 125109 (streptomycin-resistant variant M1525 [22]). Live bacterial loads in mesenteric lymph nodes (MLN), spleen and cecal content were determined by plating on MacConkey-agar (Oxoid) with respective antibiotics [21]. *Lactobacillus reuteri* ^RR^ (8*10^6^ cfu i.g.) was administered by gavage and cultured anaerobically on MRS media (Biolife; 100 µg/ml rifampicin). To enoumerate bacteria, cecal content was stained with Sytox-green and bacteria were counted in a Neubauer-chamber. Bacterial density is given as Sytox-green positive bacteria per gram cecal content.

### Ethics statement

All animal experiments were approved (license 201/2004 and 201/2007 Kantonales Veterinäramt Zürich) and performed as legally required.

### Bacteria

The streptomycin-resistant wild type strain *S*. Typhimurium (SL1344 wildtype [23]), the isogenic mutant *S*. Typhimurium^ avir^ (Δ*invG sseD*::*aphT*; *kan* ^R^ [24]) and wild type *S*. Enteritidis (M1525 [22]) were grown in LB 0.3 M NaCl as described [24]. *L. reuteri* ^RR^ [12] was isolated from our mouse colony selected on MRS media (100 µg/ml rifampicin) (Biolife) and grown anaerobically.

### Histology

HE-stained cecum cryosections were scored as described, evaluating submucosal edema, PMN infiltration, goblet cells and epithelial damage yielding a total severity score of 0-13 points [21]. 0–3 = no to minimal signs of inflammation which are not sign of a disease; this is frequently found in the cecum of conventional mice. 4–8 = moderate inflammation; 9–13 = profound inflammation.

### Statistical analysis

Statistical analysis of *Salmonella* colonization titers was performed using the exact Mann-Whitney U Test (SPSS Version 14.0). P-values less than 0.05 (2-tailed) were considered statistically significant. Pearson- and Spearman correlation coefficients for bacterial colonization levels were calculated using Graphpad Prism (Version 5.01). Other statistical analyses (Pearson correlation, Kolmogorov-Smirnov test) were performed using the statistical language and environment R (http://www.r-project.org/). To systematically detect differentially abundant OTUs in all mice and for different clustering distances, we used the R software Metastats [25].

### Bacterial DNA extraction and 16S rRNA gene specific PCR

Total DNA was extracted from cecal contents using a QIAmp DNA stool mini kit (Qiagen). Bacterial lysis was enhanced using 0.1 mm glass beads in buffer ASF and a Tissuelyzer device (5 minutes, 30 Hz; Qiagen). V5-V6 regions of bacterial 16S rRNA were amplified using primers B-V5 (5′ GCCTTGCCAGCCCGCTCAG ATT AGA TAC CCY GGT AGT CC 3′) and A-V6-TAGC (5′ GCCTCCCTCGCGCCATCAG [TAGC] ACGAGCTGACGACARCCATG 3′). The brackets contain one of the 20 different 4-mer tag identifiers [TAGC, TCGA, TCGC, TAGA, TGCA, ATCG, AGCT, AGCG, ATCT, ACGT, GATC, GCTA, GCTC, GATA, GTCA, CAGT, CTGA, CAGA, CTGT, CGTA;]. Cycling condition were as follows: 95°C, 10 min; 22 cycles of (94°C, 30 s; 57°C, 30 s; 72°C, 30 s); 72°C, 8 min; 4°C, ∞; Reaction conditions (50 µl) were as follows: 50 ng template DNA; 50 mM KCl, 10 mM Tris-HCl pH 8.3, 1,5 mM Mg^2+^, 0,2 mM dNTPs; 40 pmol of each primer, 5U of Taq DNA polymerase (Mastertaq; Eppendorf).

PCR products of different reactions were pooled, ethanol-precipitated and fragments of ∼300 bp were purified by gel electrophoresis, excised and recovered using a gel-extraction kit (Machery-Nagel). Amplicon sequencing of the PCR products was performed using a 454 FLX instrument (70×70 Picotitre plate) according to the protocol recommended by the supplier (www.454.com). PCR to detect ASF bacteria in the feces was done as described in [26].

### *E. coli* differentiation

Candidate *E. coli* strains yielding large, red colonies on MacConkey agar were typed using Enterotubes (BD Biosciences). Additionally, in some cases 16S rRNA gene sequencing was performed. The amplification was performed with extracted DNA using ”broad-range” bacterial primers fD1 and rP1 [27]. Reaction conditions were as follows: Deoxyribonucleoside triphosphates (0.25 mM), primers (1 pmol/µl each), 5UTaq-DNA polymerase (Mastertaq; Eppendorf), 50 ng of template DNA. The following cycling parameters were used: 5 min of initial denaturation at 94°C followed by 35 cycles of denaturation (1 min at 94°C), annealing (1 min at 43°C), and elongation (2 min at 72°C), with a final extension at 72°C for 7 min. Amplified PCR products were purified by gel electrophoresis and sequenced using rP1 as sequencing primer. Sequences were assigned to the RDP taxonomy using the RDP classifier (http://rdp.cme.msu.edu/; [28]).

### Quantification of Lactobacilli

Fecal samples were re-suspended in PBS and plated in appropriate dilutions on MRS agar (DE MAN, ROGOSA und SHARPE; Biolife) that supports growth of *Lactobacillus* spp. as well as *Leuconostoc* spp. and *Pediococcus* spp. Plates were incubated for 24 h in an atmosphere of 7% H_2,_ 10% CO_2_ and 83% N_2_ at 37°C in anaerobic jars.

### Reads sorting and quality filtering

The amplicon library was sequenced according to the 454 Amplicon Sequencing protocols provided by the manufacturer (Roche 454) at the McMaster University Hamilton (Canada). The sequence determination was made using GS Run Processor in Roche 454 Genome Sequencer FLX Software Package 2.0.00.22. Performance of the sequencing run was gauged using known pieces of DNA introduced in the sequencing run as DNAControl Beads. On average, 94% of reads from DNA Control Beads matched the corresponding known sequences with at least 98% accuracy over the first 200 bases, which was above the typical threshold (80% matches of 98% accuracy over 200 bases). To estimate the reliability of sample separation using our primer-tagging approach, we assessed the number of reads observed to have an illegitimate 4-mer tag (i.e., different from our set of 20 tags). The sequencing plate (including other non-analyzed samples) produced a total of 264,503 reads from which 1,339 contained a wrong tag (0.506%). Given that 256 distinct 4-mer tags are possible and that we used only 20 of these, the majority of sequencing errors in this region are detectable. Correcting for the small fraction of undetectable errors (20/256) and division by four yields a sequencing error rate of 0.137% per single nucleotide - at the position of the tag in the primer (this includes errors during primer synthesis as well as sequencing). Because most errors are actually visible as errors, the rate of unintentional ‘miscall’ of the sample is 0.043%.

We applied quality control of 454 reads in order to avoid artificial inflation of ecosystem diversity estimates [29]. Reads containing one of the exact 4 nt tag sequences were filtered with respect to their length (200 nt ≤ length ≤300 nt). Quality filtering was then applied to include only sequences containing the consensus sequence (‘ACGAGCTGACGACA[AG]CCATG’) of the V6 reverse primer and displaying at maximum one ambiguous nt ‘N’. The latter criterion has been reported as a good indicator of sequence quality for a single read [30]. We identified 5,268 reads shorter than 200 nt, 228 reads longer than 300 nt and 2,169 reads containing more than one ‘N’. After filtering, 190,728 reads remained (initial total of 197,949 reads containing the exact primer sequence and tag) and were processed as described below.

### Definition of OTUs

OTUs were defined using the complete filtered dataset, with the exception of exactly identical reads, which were made non-redundant to reduce computational complexity. Before OTU generation, we added reference sequences for subsequent taxonomic classification of OTUs; for this, we used a reference database of selected 16S rRNA gene sequences downloaded from the Greengenes database (http://greengenes.lbl.gov/Download/Sequence_Data/Greengenes_format/greengenes16SrRNAgenes.txt.gz, release 01-28-2009 [31]). In Greengenes, all entries are pre-annotated using several independent taxonomy inferences including the RDP taxonomy. Our reference database was built using full-length non-chimeric sequences with a minimum length of 1100 nt (in order to fully cover the V6 region of all entries). No archaeal sequences were included.

The alignment of non-redundant reads from all mice with the reference database was performed using the secondary-structure aware Infernal aligner (http://infernal.janelia.org/, release 1.0, [32]) and based on the 16S rRNA bacterial covariance model of the RDP database (http://rdp.cme.msu.edu/; [28]).

Before defining OTUs, we first removed reference sequences for which the alignment was not successful (Infernal bitscore <0). The alignment was then processed to include an equivalent amount of information from every read. To do so, we identified the consensus reverse primer sequence of the V6 region within the aligned sequence of *Escherichia coli* K12, as a reference. The full alignment was then trimmed from the start position (defined by the *E. coli* V6 reverse primer) and ended after 200 nt's. This also insured the limitation of the effect of pyrosequencing errors by trimming the 3′ end of each read, a region which is more sequencing-error prone (the trimmed and aligned reads length ranged from 192 to 241 nt) [29]. Using this alignment, OTUs were built by hierarchical cluster analysis at various distances (0.01, 0.03, 0.05, 0.10, 0.15 and 0.2) using the ‘complete linkage clustering’ tool of the RDP pyrosequencing pipeline (http://pyro.cme.msu.edu/ [28]).

### Taxonomy assignment

As a first step, taxonomy was predicted for all reads using the stand-alone version of the RDP classifier (http://sourceforge.net/projects/rdp-classifier, revision 2.0, [33]). Taxon predictions were considered reliable if supported by a minimum bootstrap value of 80%. In order to predict taxonomy for each OTU, we either used any reference sequences present within a cluster, or the taxonomy of the reads present in the cluster, as predicted by the RDP classifier. To increase the resolution of the prediction, we privileged any reference sequences over the reads. For each OTU, taxonomy was inferred by a simple majority vote: if more than half of the reference sequences (or reads) present within a cluster agreed on a taxon, the OTU was annotated according to this taxon. In case of conflicts, we assigned a consensus taxon to a higher phylogenetic level for which the majority vote condition was respected.

OTU distribution between the different experimental groups and predicted taxonomies were visualized as heatmaps generated by custom Python scripting and the statistical software package R (www.r-project.org).

### Chimera estimation

Deep pyrosequencing on the 454 platform has revealed extensive microbial diversity that was previously undetected with culture-dependent methods [34]. Nevertheless, the details of protocols to generate this type of data should always be carefully considered; various types of bias can be introduced at different steps. Here, sequencing was performed on pools of PCR products, thus limitations and biases of this technique have to be taken into account to interpret the results. The abundances of amplicons may not accurately reflect the relative abundances of the template DNA because of differential primer binding- and elongation-efficiencies. Moreover, during amplification, chimeric sequences can be generated.

On such short sequences, recombination points (recombination can occur from an incompletely extended primer or by template-switching [35]) are extremely difficult to detect. Recently, a new tool to filter noise and remove chimera in 454 pyrosequencing data has been published [36]. In this study, the authors suggest that because of sequencing errors, diversity estimates may be at least an order of magnitude too high. To our best knowledge, at the time of analysis, there were no available tools to detect chimera within libraries of short 454 reads. Therefore, to detect chimera we decided to compare taxonomies assigned to N-terminal and C-terminal read fragments. A read was regarded as ‘non-chimeric’ if the best hits (BLASTn) for both of its fragments had a minimum identity of 95% and a minimum bit-score of 150. These cutoffs were selected heuristically in order to insure a reasonable alignment length and a relatively high identity to the matching reference sequence. A given read was deemed chimeric when the taxonomies of the best hits of each half were clearly not congruent (i.e., differing at the phylum level). Our simple chimeric reads detection method resulted in a higher rate of detected chimera compared to the method of Quince et *al.*, 2009 (∼7% compare to ∼3% in their example) adapted from the Mallard algorithm [37], suggesting that our approach is probably stringent enough at detecting chimera [36].

### OTU abundance correlation analysis

In order to test the general hypothesis that closely related bacteria are present at similar levels in CON mice, we systematically compared the relative abundance between all OTUs detected in 9 distinct CON mice. Here, a detected OTU was defined as present in at least 6 mice (2/3). For each possible pair of OTUs, we computed the Pearson correlation coefficient of their relative abundance (number of reads normalized by the total number of reads in a given sample) in each CON mouse. To compare these results to the distance between 2 OTUs we computed identities between all considered OTUs using their representative sequences in the complete alignment (all reads and all reference sequences). An OTU's representative sequence is defined as the sequence that has the minimum sum of the square of the distances to all other sequences within that cluster.

For statistics inference, we semi-randomized our results by shuffling non-null abundances between all detected OTUs. For both distributions we plotted running medians (y-axis) with a window size of 500 data points (the window size was decreased towards the beginning and end of the distributions). The Kolmogorov-Smirnov test (one- and two-tailed) was used to compare both distributions (actual data and random data) with respect to the deviation of the running median from the random expectation. The test was computed on x-axis bins (0.1) in order to better interpret the results of the analysis. The data processing and plotting were performed using Python scripting.

## Results

### LCM mice are susceptible to *S*. Typhimurium induced gut inflammation without the need for antibiotic treatment

**Figure 1:**
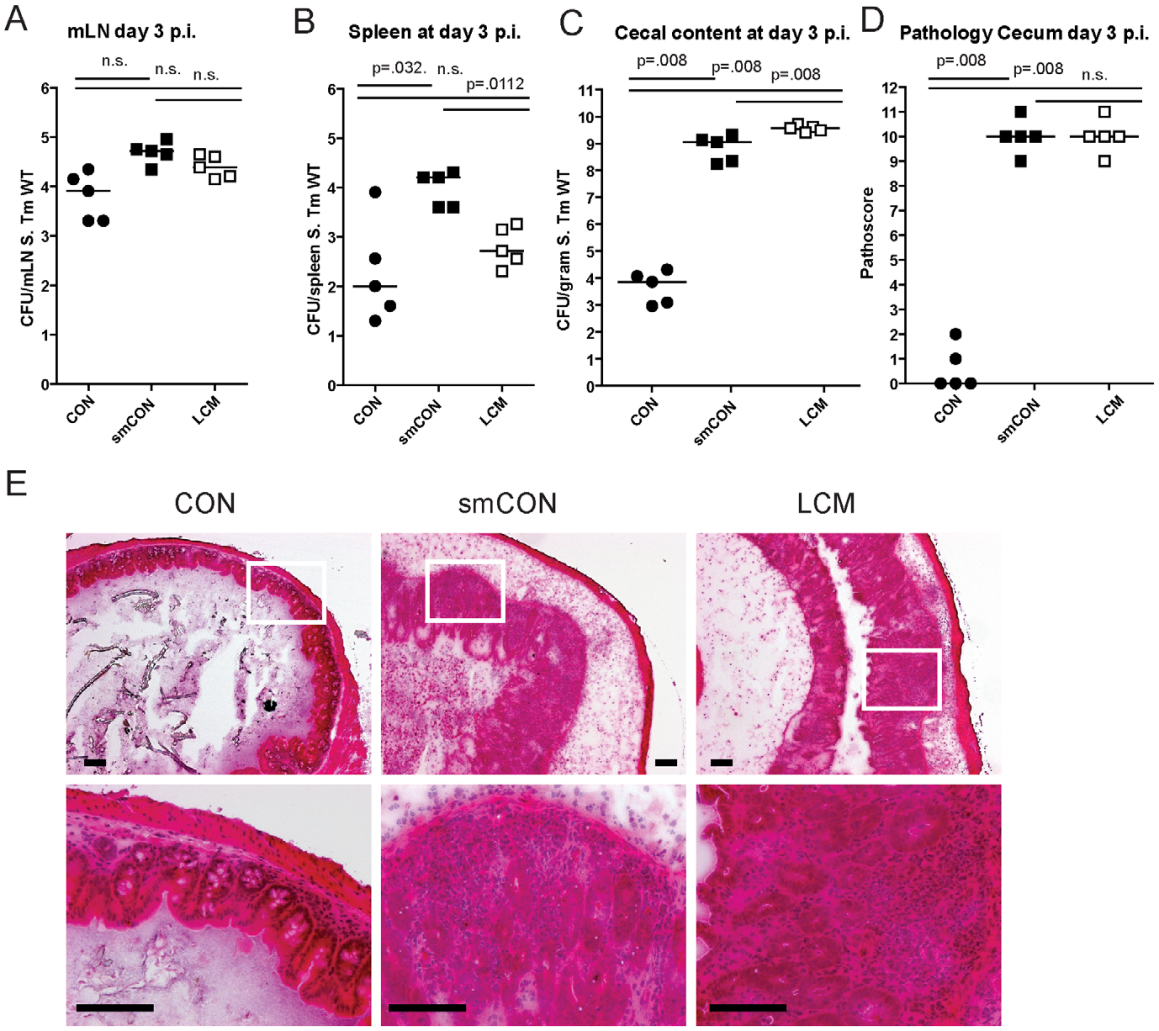
LCM mice susceptible to *S*. Typhimurium induced colitis. Groups (n = 5) of CON, streptomycin-treated mice (20 mg 24 h before infection) and LCM mice were infected with 5×10^7^ cfu *S*. Typhimurium wild type by gavage and sacrificed at day 3 postinfection. S. Typhimurium levels in the mLN (A), spleen (B) and cecal content (C). (D) Cecal pathology scored in HE-stained tissue sections (see M&M). (E) HE-stained sections of cecal tissue from indicated mice. Enlarged section (white box) is shown in the lower panel. Scale bar: 100 µm.

Germfree mice and CON mice orally treated with a single dose of antibiotic (i.e. aminoglycosides, β-lactams, vancomycin) are highly susceptible to enteric *S*. Typhimurium colonization and develop acute inflammation of the lower intestine (cecum, colon) upon oral infection [11],[18],[38]. Here, we tested susceptibility of gnotobiotic mice, associated with a standardized low complex type of gut microbiota (termed LCM), to oral *S*. Typhimurium infection. In contrast to CON mice (∼500 different bacterial strains in the gut), the gut microbiota of LCM mice includes a mixture of only 8 bacterial strains, the Altered Schaedler Flora (ASF), which are typically found in the gut of rodents [39]. In order to test whether LCM mice were susceptible to oral *S*. Typhimurium infection, we infected unmanipulated LCM mice (n = 5) by orally gavaging them with *S*. Typhimurium wild type (5×10^7^ cfu). As control, we infected age-matched groups of CON mice (n = 5) harboring a normal fully differentiated gut microbiota and CON mice pretreated with streptomycin 24 h prior to infection (smCON). All mice were sacrificed at 3 days p.i. *S*. Typhimurium titers at in the mLN and spleen were highest in smCON mice, while no difference was observed comparing CON and LCM groups (Fig. 1A,B). In keeping with previous work, the cecum of untreated CON mice was poorly colonized by *S*. Typhimurium (below 10^5^ cfu/g) while smCON mice displayed high *S*. Typhimurium levels in their gut (>10^8^ cfu/gram; p<0.05; Fig. 1C). Interestingly, LCM mice also displayed high pathogen titers in the cecum. Owing to this high-level colonization, wild type *S*. Typhimurium triggered a fulminant inflammatory response in the cecum and colon of both smCON and LCM mice, while no pathological changes could be observed in the CON mice not pretreated with antibiotics (Fig. 1D,E; Fig. S1). This demonstrates that, in contrast to normal complex type of gut microbiota, colonization of mice with a LCM gut microbiota does not confer CR against *S*. Typhimurium.

To verify that mucosal inflammation induced by *S*. Typhimurium in infected LCM mice is induced by *Salmonella*-specific virulence factors, we infected LCM mice with an avirulent mutant lacking a functional TTSS-1 and 2 (*S*. Typhimurium^avir^; 5×10^7^ cfu). Despite colonizing the gut to high titers, *S*. Typhimurium^avir^ did not cause observable signs of intestinal pathology in LCM mice, demonstrating that gut inflammation in LCM mice was triggered by the same pathogenetic mechanisms as shown for smCON mice (Fig. S2).

### Colonization resistance is transferred by re-association with a conventional gut microbiota

LCM mice, with a low complexity gut microbiota are susceptible to oral *S*. Typhimurium infection and develop severe acute colitis comparable to germfree or antibiotic-treated mice. Of note, microbiota in the cecum of LCM mice had a similar density as in CON mice (Fig. S3). These findings suggested that their gut microbiota lacks key bacterial species responsible for mediating CR. We reasoned that these protective bacteria would be transferable by co-housing LCM together with CON mice in the same cage. To test this hypothesis, we re-associated 2 groups of LCM mice (n = 2, 4) with one CON donor mouse each for 21 days. As controls, we used groups of non re-associated LCM and CON mice. We infected all animals with *S*. Typhimurium wild type (5×10^7^ cfu by oral gavage) to measure the degree of CR.

**Figure 2:**
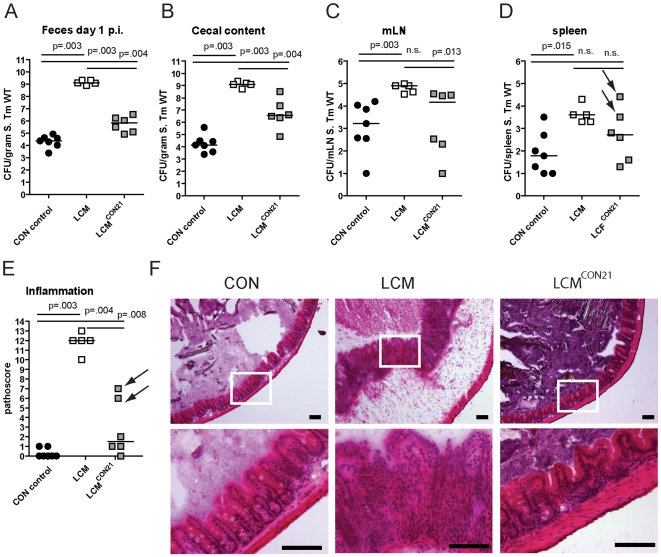
LCM gain CR by re-association with normal CON microbiota. Groups (n = 2,4) of LCM mice were re-associated with 1 CON donor each for 21 days in the same cage. Afterwards, non-reassociated LCM (control; n = 5), CON (control; n = 5) and re-associated LCM (n = 6) were infected with 5×10^7^ cfu *S*. Typhimurium wild type by gavage for 3 days. *S*. Typhimurium levels in the feces at day 1 post infection (A), cecal content (B), mLN (C), spleen (D). (E) Cecal pathology scored in HE-stained tissue sections (see M&M). (F) HE-stained sections of cecal tissue from indicated mice. Enlarged section (white box) is shown in the lower panel. Scale bar: 100 µm. Arrows point at 2 mice that developed inflammation.

Compared to unmanipulated LCM, all re-associated LCM mice had significantly lower *S*. Typhimurium loads in their feces at 1 day p.i. (Fig. 2A). 4 out of 6 animals were completely protected from *Salmonella*-colitis and did not show any signs of cecal pathology (Fig. 2E,F) while 2 out of 6 animals developed signs of inflammation (pathoscore 6 and 7) at day 3 p.i., which correlated with higher *S*. Typhimurium loads in the cecum of these mice (Fig. 2B). Systemic *S*. Typhimurium colonization appeared also slightly reduced in re-associated LCM mice (Fig. 2C,D). This revealed that CR is transferable and suggested that discrete bacterial species transferred during the 3 week re-association contributed to colonization resistance and protection from colitis.

### Microbiota analysis by high throughput amplicon-pyrosequencing

This offered the opportunity to correlate the changes in microbiota composition in the LCM mice with acquisition of colonization resistance. Protection from *Salmonella* diarrhea is conferred by bacteria entering the gut microbiota of LCM mice. To identify bacteria transferred during re-association, we analyzed gut microbiota composition by high-throughput sequencing of bacterial 16S rRNA genes. We analyzed the fecal microbiota because this non-invasive sampling method allows monitoring the microbiota of a given animal at various time points (i.e. before/after re-association or *Salmonella* infection). In contrast to other studies [2],[4],[34], we decided to sequence the 16S rRNA hypervariable regions V5 and V6 (length in *E. coli*: ∼280 bp). Several studies have shown that sequencing of different hypervariable regions or full-length 16S rRNA genes yields to comparable results [30]. Thus we reasoned that V5V6 sequencing would not lead to a major bias in microbiota composition and at the same time would allow us to fully use current pyrosequencing capacity (the average output length of the 454FLX instrument is 250 bp).

After read-quality filtering, we obtained 190,728 reads with a length between 200–300 bps in total. Among those, 50,860 were non-redundant. The frequency of chimera, using a simple identification approach was 6.9% of the total reads (13,206) and 14.7% of non-redundant reads (7,499) (Fig. S4). This chimera-frequency is relatively high considering that we probably detected only a fraction of chimeric reads using our method (materials and methods). Sequence reads were aligned with all quality-filtered sequences of our reference database generated from the Greengenes database [31] and operational taxonomic units (OTUs) were defined by hierarchical clustering at various distances, from 0.01 to 0.2. Taxonomy assignment was inferred using annotation from the reference sequences, if possible, or by predictions generated by the RDP classifier from the RDP database [28].

### Microbiota complexity differs between LCM, LCM^CON21^ and CON mice

Comparing the average number of OTUs at various distances, clearly the CON donor mice display the highest level of complexity (Tables S1, S2 and S3). We found an average of 767±233 OTUs at a Clustering Distance (CD) of 0.03 and 499±139 OTUs at a CD of 0.05 (before chimera removal: 971±290 OTUs at a distance of 0.03 and 662±186 OTUs at a CD of 0.05). Complexity of the LCM gut microbiota was, as expected, relatively low. By strain-specific PCR [26], we only detected 4 members of the ASF (ASF361, ASF457, ASF500 and ASF519; Fig. S5). However, 29±10 OTUs at a 0.03 CD, and 17±5 OTUs at a 0.05 CD were detected (before chimera removal: 38±10 OTUs at a CD of 0.03 and 23±5 OTUs at a CD of 0.05). This was expected considering the way the LCM mice were generated. LCM status was created by inoculating germfree mice with bacteria of the ASF. Afterwards, LCM mice were kept in individually ventilated cages (IVCs). During this phase, a limited number of additional species might have been acquired. This might explain why our sequence analysis detected more than 8 different phylotypes in unmanipulated LCM mice. Alternatively, the relatively high number of phylotypes could be explained by PCR artifacts or most likely by the intrinsic error rate of pyrosequencing that can lead to a severe over-estimation of microbial diversity using the 16S rRNA marker gene [29]. In LCM^CON21^ mice, we observed a significant increase in gut microbiota complexity compared to LCM mice. At a 0.03 CD, 295±34 OTUs and at a CD of 0.05, 188±23 OTUs were detected (before chimera removal: 409±60 OTUs at a CD of 0.03 and 279±45 OTUs at a CD of 0.05). However, complexity in LCM^CON21^ mice remains significantly lower than that in CON mice.

We assessed the richness (actual diversity) of our samples by calculating the Shannon index (H) and species evenness (E) as well as the Chao1 diversity estimate (Tables S1, S2 and S3). These calculations revealed that the community was clearly under-sampled; for small CDs (0.01 to 0.05), the Chao1 estimator was, for each mouse, higher than the total number of OTUs. Although under-sampling is limiting our view on the true microbial diversity, it is legitimate to use diversity measures for relative comparisons among samples. Within this context, it is interesting to ask whether, after re-association, LCM mice display similar or different species evenness E compared to the CON mice. Here, species evenness can be regarded as the equilibrium between community members; the less variation is observed between species, the higher is the E value (in other words, evenness is greatest when species are equally abundant). The E-value is defined as the ratio of the theoretically maximal Shannon-index (if all observed phylotypes were equally abundant) divided by the actual Shannon-index. For a 0.05 CD, CON mice displayed an average E-value of 0.76 compared to an average of 0.70 for the LCM^CON21^ mice (compared to E = 0.15 for LCM mice). Thus, there is no major difference between CON mice and re-associated LCM mice with respect to evenness. Hence the 21 days of co-housing were sufficient in order to adopt a relatively complex and ‘in equilibrium’ microbial gut community.

**Figure 3:**
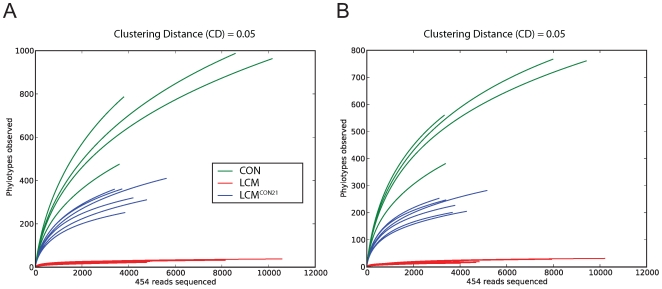
Collectors' curves of LCM, LCM^CON21^ and CON mice reveal different complexity. Collectors' curves were created for CD = 0.05 for each mouse from the total number of filtered sequences (A) or from chimera-removed sequences (B). *CON mice (green), LCM mice (red) and LCM^CON21^ mice (blue)*.

To compare species richness between the 3 different groups, rarefaction curves were created for different CDs (Fig. 3; Fig. S6). For a CD of 0.01, slopes for CON and re-associated CON mice are rather steep, revealing again a considerable under-sampling in our experiment. However, slopes for 0.05 (for re-associated LCM) and 0.1 CD (for CON) seem to reach saturation, suggesting that for this level of analysis, the sampling was sufficiently complete. Therefore we decided to perform OTU analyses using a CD higher or equal to 0.05. This CD is in accordance with a recent report advising a stringent quality-based filtering of 16S- 454 reads and the use of a clustering threshold no greater than 97% [29]. Given the clear under-sampling and the sequencing strategy applied here, a species-level analysis is not conclusive and we decided to focus our further analysis at a higher taxonomic level (from the Family up to the Phylum).

### Analysis of differences between CON and LCM^CON21^ gut microbiota

We next analyzed qualitative changes in microbiota composition during re-association. In particular, we focused at identifying which OTUs were transferred from the CON donor mice to the LCM recipients within 21 days. Those bacteria may contribute to protection against *S*. Typhimurium colonization.

In order to predict taxonomy for each OTU, we used either the reference sequence taxonomy information present within an OTU-cluster, if any, or the reads taxonomy predicted by the RDP classifier. To test if the taxonomy assignment via reference sequences provided a more resolved taxonomy, we compared taxonomy resolution obtained via reference sequences and via RDP-classifier annotated reads for OTUs which contained both reads and reference sequences (Fig. S7). For different CD and different taxon levels, the reference taxonomy always provided better taxonomic resolution from the phylum level (taxon_1) down to the genus level (taxon_5).

**Figure 4:**
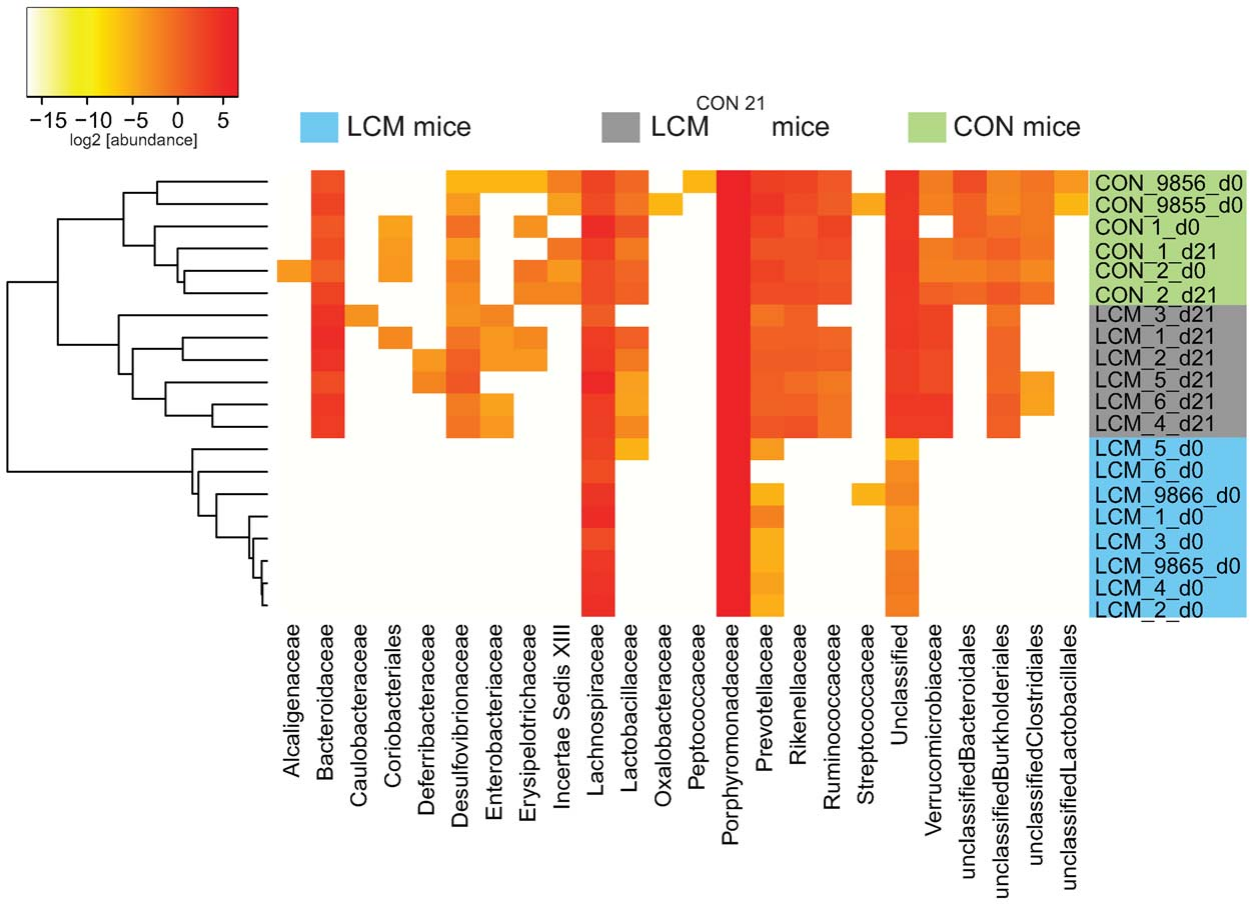
Heatmap showing OTU's distribution in different groups. Fecal microbiota of unmanipulated LCM mice was analyzed at day 0 (n = 8). 6 of these LCM mice (LCM_1 to LCM_6; blue) were conventionalized in two groups with 2 different CON-donors (CON_1 and CON_2; green) and fecal microbiota analyzed at day 21 (LCM_x_d21; grey). OTUs (CD = 0.05) were sorted according to taxon_4 (Family level; x-axis) and average clustering was performed on Euclidean distances calculated between abundance profiles for each mouse and every time-point sampled. Red color indicates high abundance (Log_2_), yellow color low abundance. CON_9855_d0 and CON_9856_d0 and LCM_9865_d0 and LCM_9866_d0 are 2 additional CON or LCM mice, respectively sampled only at day 0.

Euclidean distances between relative abundance profiles were computed for each mouse and every time-point sampled. Hierarchical clustering (average method) of all mice for taxon_2 (class) taxon_3 (order) and taxon_4 (family) were visualized on distinct heatmaps (Fig. 4; Fig. S8A,B). All CON mice (day 0 and day 21) clustered together as well as all the LCM mice before re-association. Additionally, we included two unmanipulated CON mice (donor 9855 and 9856) that were only sampled at one time-point to provide more samples of independent CON mice from the same mouse colony (n = 4 in total).

All samples of LCM mice from day 0 (before re-association) were highly similar and clustered together. The highest identity (determined by BLAST, all against all) between the V5V6 regions of the 8 different ASF members is of 93% (data not shown); therefore it is theoretically possible, for a small clustering distance, to detect each ASF species by our sequencing and taxonomy inference approach. Seven OTUs were systematically detected in the LCM mice, all assigned to the Firmicutes and Bacteroidetes phyla. Thus, we assume that the most abundant species in the feces of LCM-mice are ASF500 (Firmicutes; Clostridia; Clostridiales; Lachnospiraceae; unclassified_Lachnospiraceae) and ASF519 (Bacteroidetes; Bacteroidetes; Bacteroidales; Porphyromonadaceae; Parabacteroides;). Abundance of ASF strains in different mice can be influenced by various factors [26],[40]. Hence, the sampling depth could explain the non-detection of the other ASF members, which were most probably less abundant.

### Gamma-Proteobacteria as indicators of susceptibility and resistance to *Salmonella*-infection

The qualitative microbiota analysis revealed that within the 21 days of re-association, bacteria from all detected phyla in the CON donor mice were transferred (Fig. 4; Fig. S8A,B). However, the gut microbiota of LCM^CON21^ was significantly less complex than that of CON mice, suggesting that the microbiota might also differ on a qualitative basis. This might be causally linked to the increased susceptibility to *Salmonella* infection. Thus, we compared the microbiota of CON and LCM^CON21^ with respect to lack or enrichment of specific clusters of bacteria (i.e. on order or family level). We analyzed which OTUs were significantly over- or underrepresented comparing LCM^CON21^ and CON mice. Interestingly, among others, OTUs assigned to the family of the Enterobacteriaceae were enriched in LCM^CON21^ mice, as compared to CON mice (Fig. 4; Dataset S1). Since *Salmonella* Typhimurium is also a member of the Enterobacteriaceae, the enrichment of such close relatives in LCM^CON21^ mice might be an indicator of favorable growth conditions for this type of bacteria.

**Figure 5:**
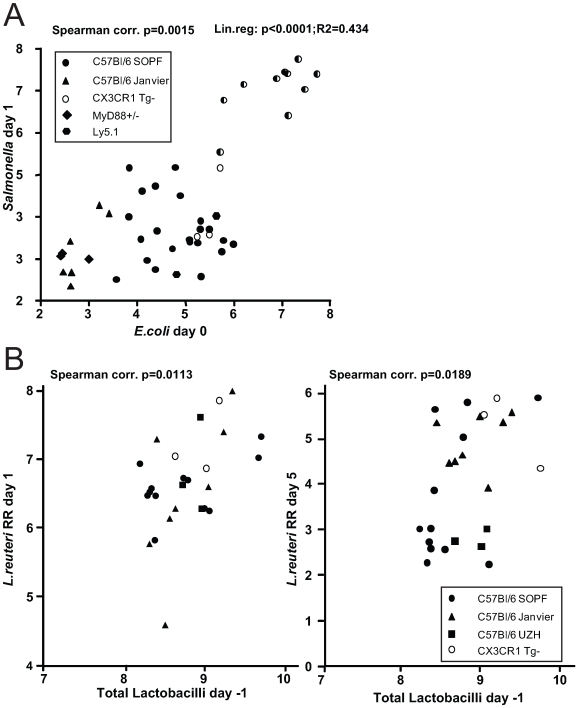
Infection experiments in conventional mice reveal correlation of bacterial infectivity with the prevalence of related species. (A) Groups normal unmanipulated CON mice (6-12 weeks; symbols indicate different sources) were infected with 5×10^7^ cfu *S*. Enteritidis wild type by gavage. Fecal *E. coli* titres before infection were determined (x-axis; Log_10_ cfu/g). 1 day post infection, *S*. Enteritidis titres in the feces were determined (y-axis; Log_10_ cfu/g). Spearman and linear correlation were calculated (p = 0.0015; p<0.0001). The degree of gut inflammation was determined in the infected mice. Half-filled symbols indicate mice with inflammation score ≥4. (B) Groups normal unmanipulated CON mice (6-12 weeks; symbols indicate different sources) were infected with 5×10^7^ *Lactobacillus reuteri* ^RR^ (rifampicin-resistant) by gavage. Fecal levels of Lactobacilli were determined on MRS agar and plotted against fecal *Lactobacillus reuteri* ^RR^ titers at day 1 (left) and 5 (right) postinfection.

This finding prompted us to investigate, whether there is a positive correlation between the abundance of Enterobacteriaceae (i.e. *E. coli*) and the susceptibility to *Salmonella* infection. We have previously observed that C57Bl/6 mice obtained from different sources (commercial breeders, other laboratories) exhibit differential degrees of CR against *Salmonella*. To analyze whether CR is linked to different *E. coli* titres, we defined fecal *E. coli* levels of mice from five different breedings (C57Bl6 background from our animal facility and others) before infecting them with *S.* Enteritidis wild type by oral gavage (5×10^7^ cfu; no antibiotic-treatment). We used *S*. Enteritidis because pilot experiments in our laboratory had shown that this serovar generally leads to a higher disease incidence (colitis at day 4 after oral infection) in non-antibiotic-treated mice, than *S*. Typhimurium. *E. coli* is readily differentiated from other Enterobacteriaceae by colony color and morphology on MacConkey agar (see Materials and Methods for typing details). One day after infection, we determined fecal *S*. Enteritidis titers by plating. The mice were sacrificed at day 4 postinfection and we analyzed *S*. Enteritidis titers at systemic sites, in the intestine as well as cecal pathology (Fig. 5A; Fig. S9). Indeed, we observed a positive linear correlation between fecal *E. coli* levels before infection, *S*. Enteritidis colonization efficiency (r^2^ = 0.434: Spearman p = 0.0015). If *S*. Enteritidis titres were above 1.5×10^5^ cfu/g feces at day 1 p.i., mice developed colitis at day 4 p.i. This suggests that *E. coli* titres may predict whether mice are susceptible to *Salmonella* induced gut inflammation.

### Higher levels of Lactobacilli predict higher intestinal colonization with a commensal *L. reuteri* ^RR^ after oral inoculation

We observed that higher *E. coli* levels positively correlate with increased *Salmonella* infectivity. This might be due to the close relatedness of these two species as they might have similar environmental requirements. Thus, we hypothesized that the same principle might apply for other intestinal bacteria. We tested this hypothesis using *Lactobacillus reuteri* ^RR^, a rifampicin-resistant isolate from our mouse colony that can be specifically detected by culture [12].

We determined whether higher titres of intestinal Lactobacilli would correlate with increased gut colonization by *Lactobacillus reuteri* ^RR^ upon oral gavage. Lactobacilli are Gram-positive, of low G+C content, non-spore-forming, aerotolerant anaerobes and can be differentiated on selective media (i.e. MRS-agar). We determined fecal levels of Lactobacilli of mice from different sources and subsequently infected them with *Lactobacillus reuteri* ^RR^ (10^7^ cfu by oral gavage). 1 and 5 days post infection we determined *Lactobacillus reuteri* ^RR^ titres in the feces. Indeed, we found significantly enhanced colonization of *Lactobacillus reuteri* ^RR^ in mice with higher titres of *Lactobacilli* (Fig. 5B). This suggests that, like in the case of *E. coli* and *Salmonella*, higher levels of Lactobacilli correlate with increased colonization efficiency by a commensal *Lactobacillus* strain.

### Closely related phylotypes generally display significantly correlated abundances in the intestine

**Figure 6:**
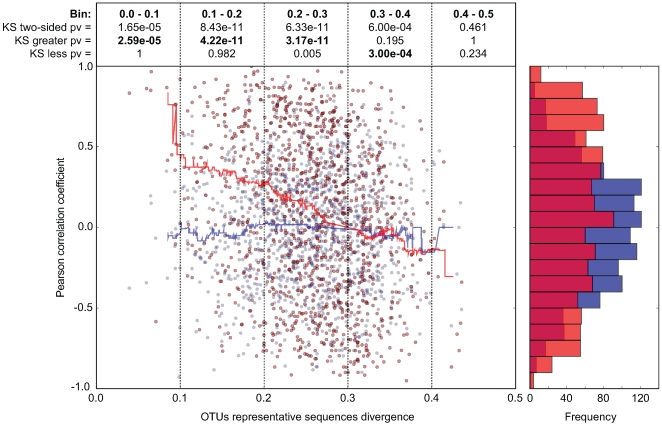
In CON mice, related bacterial lineages are preferentially observed together (quantitative co-occurrence). For all possible pairs of detected OTUs (i.e. present in at least 2/3 of the analyzed mice; CD = 0.2; each dot in the graph represents an OTU-pair), abundance correlations (y-axis, Pearson) were computed from abundance measurements in 9 distinct CON mice, and plotted against the molecular divergence between their representative 16S sequences (x-axis). The latter distances between representative sequences were computed using sequence identities as defined by the complete multiple alignment of all reads and all reference sequences. For hypothesis testing, we compared the data distribution (red) to a matched random distribution of OTU abundances generated by shuffling non-null OTU abundances between all OTUs (blue). Running medians are represented in the corresponding color. The Pearson correlation coefficient is −0.248 (p-value = 7.10e-18) for the actual data, and −0.017 (p-value = 0.563) for the randomized data. We compared the deviation of the actual data on the y-axis (Pearson correlation) from the distribution of the randomized data using the Kolmogorov-Smirnov test. Both two-sided and one-sided hypotheses (greater and less) were tested for each bin of 0.1 on the x-axis (0.0-0.1; 0.1–0.1; 0.2–0.3; 0.3–0.4; 0.4–0.5). Results are indicated in boxes in the upper part of the graph. Pv = P-value; ‘KS two-sided pv’ indicates whether there is a significant difference between the distribution of the data (red dots) and the distribution of the randomized data (blue dots); ‘KS greater pv’ indicates whether the Pearson correlation coefficient of the data (red dots) is significantly higher compared to the random background in a given bin (blue dots). ‘KS less pv’ indicates whether the Pearson correlation coefficient of the data (red dots) is significantly lower compared to the random background in (blue dots) in a given bin. The histogram on the right side of the graph represents the cumulative frequencies of the binned Pearson correlation coefficient data.

In order to investigate whether our observations with Enterobacteriaceae and Lactobacillaceae correspond to a more universal phenomenon that applies to closely related bacterial groups in general, we performed a systematic abundance correlation analysis between OTUs detected in 9 distinct CON mice (Fig. 6; Fig. S10). We limited our analysis to OTUs detected in at least 6 mice in order to lower the under-sampling bias in our 454 sequence data. Upon examination of OTUs defined at various CDs, we found that closely related phylotypes (i.e. 0<reads divergence<0.2) generally display significantly correlated abundances (co-occurrence), more so than distantly related phylotypes. In summary, our results indicate that the invasion-success of novel species into a complex gut microbiota might be predetermined by the presence of closely related species or by factors that also influence the abundance of closely related species in this ecosystem.

## Discussion

### LCM mice as model for investigating the mechanisms of CR

It has been known for a long time, that the normal gut microbiota plays a key role in protection from infection with pathogenic bacteria. Germfree mice lack CR and thus are highly susceptible to infections with various pathogens [41]. They regain CR upon conventionalization with a normal microbiota [42]. This process has been studied extensively in the 1970s and 1980s; these earlier studies mainly addressed the question, which parts of the complex gut microbiota play a role in ‘conventionalization’ and inhibition of pathogen growth [43],[44],[45]. Due to technical limitations at that time, the studies were confined to the analysis of cultivated bacteria.

In contrast to this earlier work, we use LCM mice that are colonized with a stable, low-complexity gut microbiota being composed of typical gut bacteria i.e. *Bacteroides* spp., *Clostridium* spp., *Mucispirillum* spp. and Lactobacilli (ASF361, ASF457, ASF500 and ASF519) as starting point for the re-association studies. We show that LCM mice, despite being colonized with a numerically dense gut microbiota, are still susceptible to *Salmonella* gut infection and colitis. This system represents major advantages over the use of germfree mice. First of all, the maintenance of LCM mice is by far less extensive than that of germfree mice. We have maintained a colony of LCM mice for 24 months in IVC cages without significantly altering complexity of their gut microbiota. Therefore, LCM mice harbor ‘typical’ gut bacteria which, to some extent, protects against contamination with environmental bacteria, meaning that their gut ecosystem is somewhat normalized. Secondly, compared to germfree mice, the gut mucosal immune system and innate defense is partially normalized. Consequently, *Salmonella*-induced intestinal pathology is milder in LCM that in germfree mice (this work and [11]). However, the LCM gut microbiota apparently lacks certain parts of the conventional gut microbiota important for protection against infection with enteropathogens (i.e. *Salmonella*, *E. coli*). Thus, LCM mice represent a very useful system to screen for protective bacteria and characterize the mode of protection.

What mechanisms underlie protection against enteropathogens by the gut microbiota? Host factors induced by bacterial colonization could be one mediator of CR. The gut microbiota instructs and shapes the mucosal innate and adaptive immunity and keeps the host in a defense-competent state [46],[47],[48]. Alternatively the bacteria forming the gut ecosystem directly suppress pathogen growth. This could be mediated by blocking of pathogen receptor sites or the production of antibacterial substances and metabolic by-products like short-chain fatty acids (SCFA) [49],[50],[51],[52],[53]. Moreover, conventionalization also involves drastic changes in intestinal physiology, such as decrease of relative cecal size, free nutrient depletion, oxygen limitation and lowering of the redox potential [54],[55],[56]. The intestinal microbiota consists to the greatest part of obligate anaerobic and extremely oxygen-sensitive bacteria [57] and oxygen tension in the gut decreases gradually from stomach to rectum while bacterial density increases [58]. These conditions keep colonization levels of facultative aerobic bacteria, which comprise most enteropathogens, relatively low [57],[59].

To date, the key bacteria inducing CR have not been unambiguously identified. Rolf Freter and coworkers aimed at identifying single strains that accomplish conventionalization of germfree mice. He demonstrated that a collection of 95 anaerobic intestinal isolates or even a combination of Clostridia and *Lactobacillus* spp. isolates is sufficient to restore CR [43],[45]. To our knowledge, these ‘CR-mediators’ were never further described or characterized in detail. Since this would be a critical step towards understanding the molecular basis of CR, isolation and characterization of ‘CR-mediators’ will be subject of future analyses. To this end, LCM mice will be a useful tool.

### Microbiota analysis during conventionalization

We analyzed microbiota changes during conventionalization of LCM mice using deep sequencing of 16S rRNA genes. This extends earlier studies that have focused only on culturable bacteria, to non-culturable strains. It is assumed that LCM mice pick up fecal bacteria from the CON donor mouse by coprophagy. The efficiency of microbiota transfer by coprophagy may be questionable, since a great part of conventional gut microbiota is extremely oxygen sensitive. Still, we found that representatives of all five major eubacterial phyla typically present in the mammalian gut were transferred to LCM mice within 21 days. Although microbiota complexity drastically increased within 21 days of conventionalization, it was still significantly lower than in CON mice. Interestingly, in the same way as microbiota complexity, CR of LCM^CON21^ mice against *S*. Typhimurium was at a somewhat intermediate level. Still, LCM^CON21^ mice were, at least partially, protected from *Salmonella*-induced gut inflammation.

Overall complexity of LCM^CON21^ mice was not restored to the levels of CON mice, suggesting that conventionalization takes longer than 21 days to reach a high-density equilibrium state. For example, relatively few members of the Firmicutes and a high number of Verrucomicrobia were detected. Conventionalization is proposed to be a process of ecologic succession whereby the relative composition of the microbiota constantly changes, a sequence that mirrors microbiota-colonization after birth [60]. Alternatively, as the Firmicutes branch comprises most of the extremely oxygen-sensitive species, it is conceivable that they might be transferred less efficiently, or, only after oxygen tension in the gut is low enough to allow growth. It would be very interesting to analyze microbiota composition in fecal samples of LCM mice at different time points during conventionalization and also extend the analysis to longer time points beyond 21 days.

### Levels of close relatives predict bacterial infectivity

Since LCM^CON21^ mice have partially gained CR during the 21 days re-association period, we aimed at identifying certain protective bacterial species, which are absent in LCM mice. Although microbiota complexity in LCM^CON21^ mice was too high for unequivocal species identification, comparative microbiota analysis detected an enrichment of Enterobacteriaceae in LCM^CON21^ mice. We concluded that this group of bacteria does not mediate CR (‘CR-mediator’) but may rather indicate the level of CR (‘CR-indicator’). Upon screening a variety of conventional mice from different sources, we observed that higher *E. coli* titers positively correlated with *Salmonella* infectivity. Thus, *E. coli* can be regarded as ‘CR-indicators’ for *Salmonella* infection. Higher concentrations or diversity of Enterobactericeae can be indicative for alleviated CR [61],[62]. This may explain why infection with ‘CR-indicator’ *E. coli* strains has been previously used as a method to judge the intensity of CR [63]. *E. coli* and *Salmonella* spp. are very close phylogenetic relatives. Strikingly, *E. coli* levels also correlated with susceptibility to *Salmonella*-induced colitis. When *E. coli* and concomitant *S*. Typhimurium levels were above 10^6^ cfu/g at day 1 post pathogen infection, mice reliably developed gut inflammation. Interestingly, we found a similar correlation between the level of intrinsic Lactobacilli and the colonization levels of orally inoculated *Lactobacillus reuteri* ^RR^ strain. Therefore, we speculated that the finding could be a general principle that applies to closely related bacterial groups in the intestinal ecosystem.

How can closely related species actually coexist in the same ecosystem? In theory, closely related species could occupy the same niche in the intestine although they had similar nutrient requirements or share the same adhesion receptors. However, in praxis, species A will perform slightly better than species B, which would lead to out-competition and elimination of B. Alternatively, species B could switch to the use of a different available nutrient source (or receptor) and coexist with species A in the same ecosystem. This principle has been demonstrated in case of *E. coli*. Different commensal *E. coli* strains can coexist in the intestine by using different nutrients [64].

But how is colonization level of a certain species A connected to the colonization efficiency of its close relative B? This might be explained by the fact that the same global selective pressure acts on both species. This global pressure could be the presence of a third species C that inhibits both A and B (i.e. by inhibitor production). Alternatively, A and B might have the same requirements of oxygen or the same sensitivity to antimicrobial peptides that only allows the bacteria to grow at a certain, defined density. This correlation would only be maintained, if none of the two strains produced a direct inhibitor against the other species (i.e. colicin, nisin, metabolites). Taken together, this principle suggested for Enterobacteriaceae and Lactobacillaceae might also apply for other bacterial groups, sharing common growth requirements.

### General implications

Our data suggest, that subtle fluctuations in intestinal ecosystem composition between individuals might partly explain their differential susceptibility to gut infections or probiotic therapy. This knowledge could be exploited for screens of the human population to identify certain risk-or susceptibility groups. This would then enable the correlation of these data to other parameters (lifestyle, age, gender, nutrition). The existence of a highly dynamic niche for growth of Enterobacteriaceae, varying between different individuals, might reflect the differential susceptibility to gut infections within the human population. Some patients might have suffered from insults that induce a transient ‘out of equilibrium’ state of the microbiota that renders it less protective. Such conditions could be nutrient deficiencies, stress, illness or a history of antibiotic treatment. Screening of people at risk (elderly, immune-suppressed) might thus help in early disease prevention and potentially enable more targeted use of antibiotics.

## Supporting Information

Figure S1 LCM and smCON mice develop inflammation of the cecum and colon after *S*. Tm infection. HE-stained tissue cross sections (see M&M) of the cecum, proximal and distal colon of (A) a naïve CON, (B), a smCON mouse at day 3 post infection with *S*. Tm wild type and (C), LCM mouse at day 3 post infection with *S*. Tm wild type. Enlarged section (black box) is shown in the right panels. Scale bar: 50 µm.
(3.21 MB PDF)

Figure S2 Avirulent *S*. Typhimurium do not induce inflammation in LCM mice. Groups (n = 5) of LCM mice were infected for 3 days with *S*. Typhimurium wild type or the avirulent mutant *S*. Typhimurium^avir^ (Δ*invG*; *sseD*::*aphT*). *S*. Typhimurium levels in the feces at day 1 post infection (A), cecal content (B), mLN (C), spleen (D). (E) Cecal pathology scored in HE-stained tissue sections (see M&M). (F) HE-stained sections of cecal tissue from indicated mice. Enlarged section (white box) is shown in the lower panel. Scale bar: 100 µm.
(0.50 MB PDF)

Figure S3 Microbiota density in CON and LCM mice. (A) Cecal content of CON and LCM mice was stained with Sytox-green and bacteria were counted in a Neubauer-chamber. Bacterial density is given as Sytox-green positive bacteria per gram cecal content. (B) Representative confocal fluorescence microscopy images of cecum tissue sections from the mice shown in (A). Nuclei and bacterial DNA are stained by Sytox-green (green), the epithelial brush border actin by phalloidin-Alexa-647 (blue). Scale bar: 50µm.
(0.49 MB PDF)

Figure S4 Example for a chimeric sequence read obtained by pyrosequencing. The figure depicts an example for a chimeric V5-V6 read. The mismatches of the chimeric read to the best BLASTn hits (best bit-score) using either a non-redundant (nr) Bacteroidetes database (a) or a nr Firmicutes database (b). Using our chimera detection approach, the V5 region of the depicted read was predicted as Firmicutes and its V6 region as Bacteroidetes.
(0.08 MB PDF)

Figure S5 Detection of ASF bacteria in LCM mice by PCR. Fecal bacterial DNA was extracted from LCM mice and used as template for ASF-strain specific PCR. Strain specific PCR for ASF356 (417 bp), ASF360 (131 bp), ASF361 (182 bp), ASF457 (95 bp), ASF492 (167 bp), ASF500 (285 bp), ASF502 (427 bp), and ASF519 (429 bp) (expected sizes shown in parentheses) was performed as described [26]. Linearized plasmids as positive control, containing the 16SrRNA gene of each ASF strain (A) or fecal bacterial DNA was used as template (B). Only strains ASF361, ASF457, ASF500 and ASF519 could be detected in the feces of LCM mice (here only 1 representative PCR result shown).
(0.02 MB PDF)

Figure S6 Collectors' curves of LCM, LCM^CON21^ and CON mice reveal different complexity. Collectors' curves were created for different CD (0.1; 0.03; 0.01) for each mouse from the total number of filtered sequences (A) or from chimera-removed sequences (B). CON mice (green), LCM mice (red) and LCM^CON21^ mice (blue).
(0.68 MB PDF)

Figure S7 Taxonomy resolution obtained via reference sequences and via RDP-classifier. OTU taxonomy assignment was inferred preferentially using reference sequences annotations (if present in the cluster) or using RDP classifier predictions on the 454 reads. For OTUs containing both reference sequences and reads, we compared the resolution of taxonomies assigned by majority vote via both approaches. For each clustering distance tested and for all taxon levels, a more resolved taxonomy was obtained using the reference sequences annotations.
(0.10 MB PDF)

Figure S8 Heatmap showing OTU's distribution in different groups at different phylogenic resolutions. Analysis of fecal microbiota of the mice shown in Fig. 4. Fecal microbiota of unmanipulated LCM mice was analyzed at day 0 (n = 8). 6 of these LCM mice (LCM_1 to LCM_6; blue) were conventionalized in two groups with 2 different CON-donors (CON_1 and CON_2; green) and fecal microbiota analyzed at day 21 (LCM_x_d21; grey). (A) OTUs were sorted according to taxon_2 (class level; X-axis) of (B) according to taxon_3 (order level; X-axix) and average clustering was performed on Euclidean distances calculated between abundance profiles for each mouse and every time-point sampled. Red color indicates high abundance (Log_2_), yellow color low abundance. CON_9855_d0 and CON_9856_d0 and LCM_9865_d0 and LCM_9866_d0are 2 additional CON or LCM mice, respectively sampled only at day 0.
(0.15 MB PDF)

Figure S9 Development of *Salmonella*-induced gut inflammation in mice with differential fecal *E. coli* titres. Groups of normal unmanipulated CON mice (6–12 weeks; symbols indicate different sources; x-axis) were infected with 5×10^7^ cfu *S*. Enteritidis wild type by gavage and sacrificed at day 4 p.i. (see Fig. 5A). Cecal pathology of mice was analyzed in HE-stained tissue sections (see Materials and Methods).
(0.14 MB PDF)

Figure S10 In CON mice, related bacterial lineages are preferentially observed together (quantitative co-occurrence). For all possible pairs of detected OTUs (i.e. present in 2/3 of the analyzed mice; A, CD = 0.1 and B, CD = 0.05; each dot in the graph represents an OTU-pair), abundance correlations (y-axis, Pearson) were computed from abundance measurements in 9 distinct CON mice, and plotted against the molecular divergence between their representative 16S sequences (x-axis). Running medians are represented in the corresponding color. We compared the deviation of the actual data on the y-axis (Pearson correlation) from the distribution of the randomized data using the Kolmogorov-Smirnov test. Both two-sided and one-sided hypotheses (greater and less) were tested for each bin of 0.1 on the x-axis (0.0–0.1; 0.1–0.1; 0.2–0.3; 0.3–0.4). Results are indicated in boxes in the upper part of each graph. Pv = P-value; ‘KS two-sided pv’ indicates whether there is a significant difference between the distribution of the data (red dots) and the distribution of the randomized data (blue dots); ‘KS greater pv’ indicates whether the Pearson correlation coefficient of the data (red dots) is significantly higher compared to the random background in (blue dots) in a given bin. ‘KS less pv’ indicates whether the Pearson correlation coefficient of the data (red dots) is significantly lower compared to the random background in (blue dots) in a given bin. The histogram on the right side of the graph represents the cumulative frequencies of the binned Pearson correlation coefficient data.
(6.78 MB PDF)

Table S1 Parameters of microbial complexity of CON-donors day 0 (n = 4).
(0.06 MB DOC)

Table S2 Parameters of microbial complexity of LCM-recipients day 0 (n = 8).
(0.03 MB DOC)

Table S3 Parameters of microbial complexity of LCM-recipients day 21 (n = 6).
(0.03 MB DOC)

Dataset S1 Comparative OTU abundance analysis of LCFd21 and CON mice showing differentially abundant OTU's at different Clustering Distances (ClustD).
(0.07 MB XLS)
