# Supplementary material for: Like Will to Like: Abundances of Closely Related Species Can Predict Susceptibility to Intestinal Colonization by Pathogenic and Commensal Bacteria
Source: PLoS Pathog. 2010 Jan 8;6(1):e1000711. doi: 10.1371/journal.ppat.1000711 (PMC2796170; doi:10.1371/journal.ppat.1000711)

Stecher, Chaffron, Käppeli et *al.* Figure S1

LCM and smCON mice develop inflammation of the cecum and colon after *S.*Tm infection

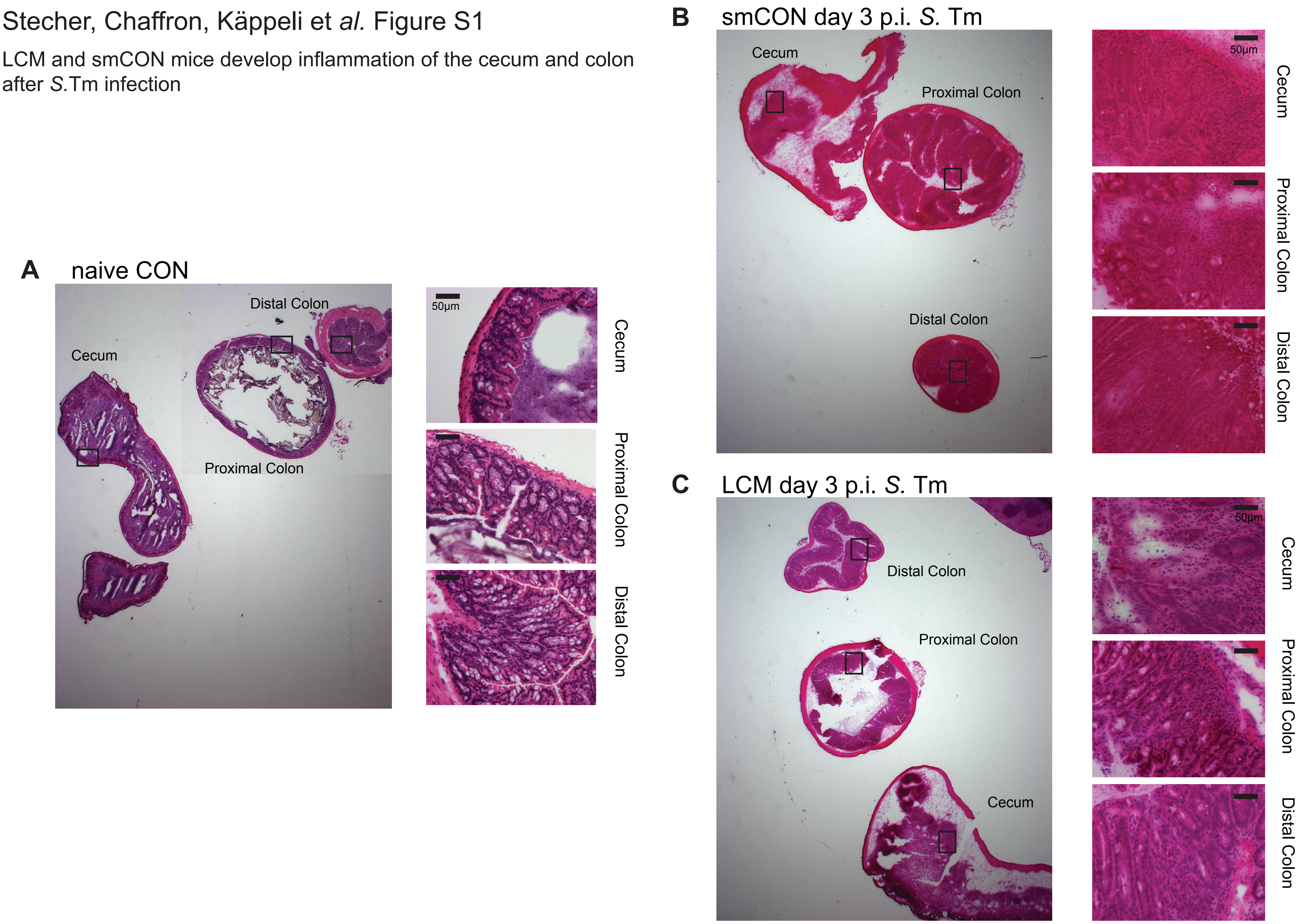

Supplement: Figure S1 — LCM and smCON mice develop inflammation of the cecum and colon after S. Tm infection. HE-stained tissue cross sections (see M&M) of the cecum, proximal and distal colon of (A) a naïve CON, (B), a smCON mouse at day 3 post infection with S. Tm wild type and (C), LCM mouse at day 3 post infection with S. Tm wild type. Enlarged section (black box) is shown in the right panels. Scale bar: 50 µm. (3.21 MB PDF) [file ppat.1000711.s001.pdf]
