# Supplementary material for: Like Will to Like: Abundances of Closely Related Species Can Predict Susceptibility to Intestinal Colonization by Pathogenic and Commensal Bacteria
Source: PLoS Pathog. 2010 Jan 8;6(1):e1000711. doi: 10.1371/journal.ppat.1000711 (PMC2796170; doi:10.1371/journal.ppat.1000711)

Stecher et *al.* Figure S2

Salmonella induced gut inflammation in LCM mice is triggered by the same mechanisms as in smCON

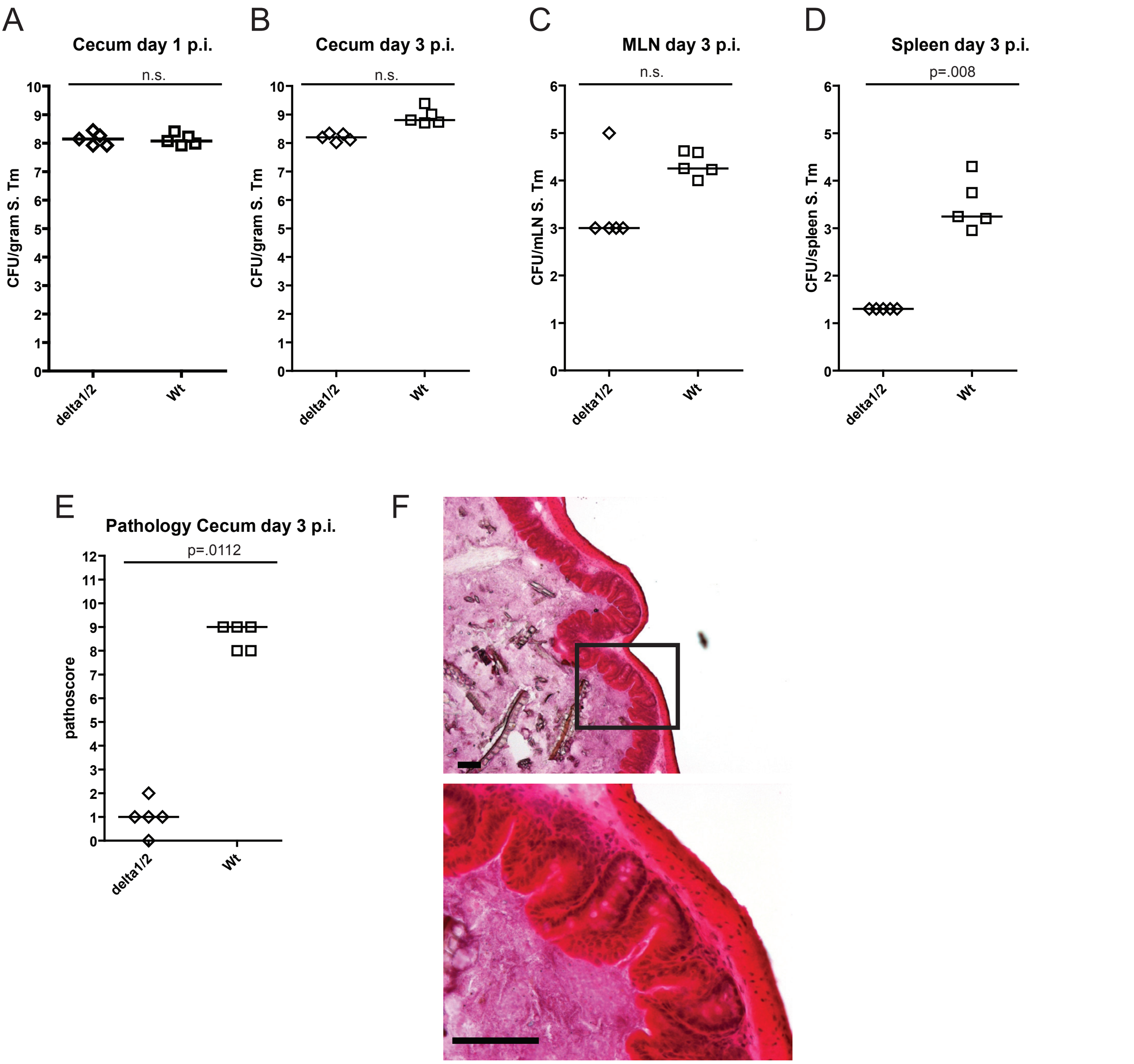

Supplement: Figure S2 — Avirulent S. Typhimurium do not induce inflammation in LCM mice. Groups (n = 5) of LCM mice were infected for 3 days with S. Typhimurium wild type or the avirulent mutant S. Typhimuriumavir (ΔinvG; sseD::aphT). S. Typhimurium levels in the feces at day 1 post infection (A), cecal content (B), mLN (C), spleen (D). (E) Cecal pathology scored in HE-stained tissue sections (see M&M). (F) HE-stained sections of cecal tissue from indicated mice. Enlarged section (white box) is shown in the lower panel. Scale bar: 100 µm. (0.50 MB PDF) [file ppat.1000711.s002.pdf]
