# Supplementary figures and images for: Like Will to Like: Abundances of Closely Related Species Can Predict Susceptibility to Intestinal Colonization by Pathogenic and Commensal Bacteria
Source: PLoS Pathog. 2010 Jan 8;6(1):e1000711. doi: 10.1371/journal.ppat.1000711 (PMC2796170; doi:10.1371/journal.ppat.1000711)

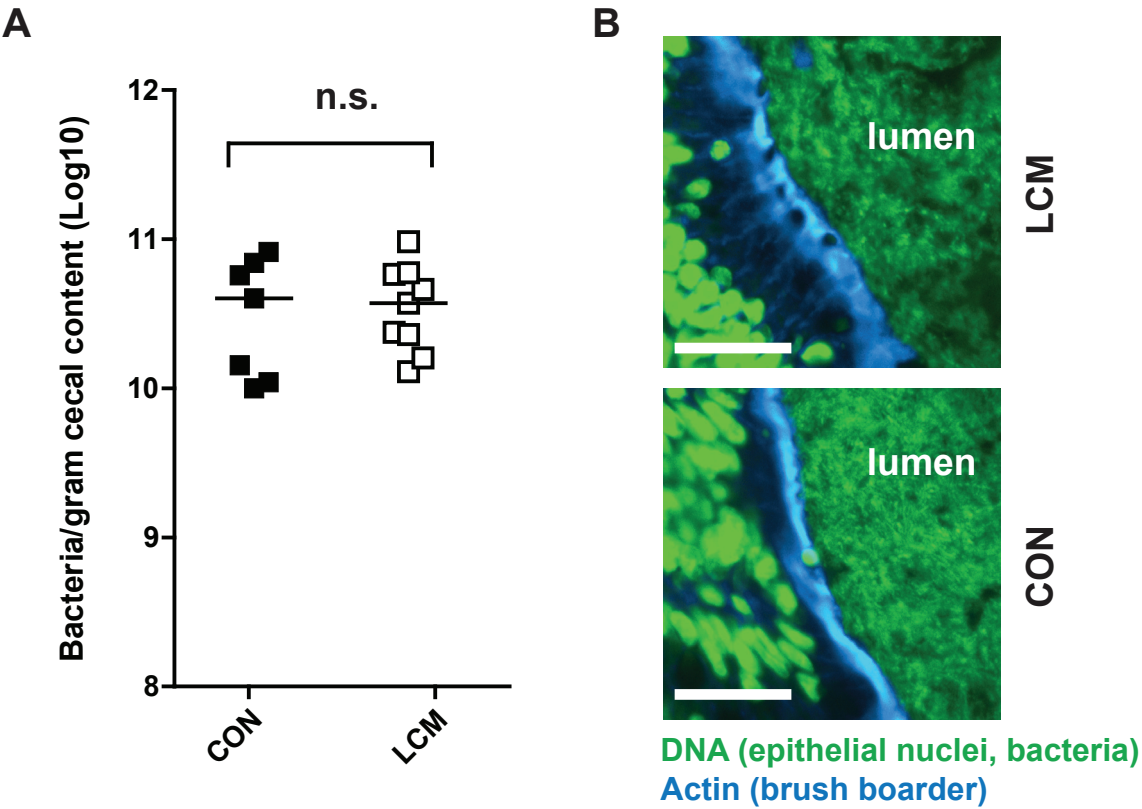

Supplement: Figure S3 — Microbiota density in CON and LCM mice. (A) Cecal content of CON and LCM mice was stained with Sytox-green and bacteria were counted in a Neubauer-chamber. Bacterial density is given as Sytox-green positive bacteria per gram cecal content. (B) Representative confocal fluorescence microscopy images of cecum tissue sections from the mice shown in (A). Nuclei and bacterial DNA are stained by Sytox-green (green), the epithelial brush border actin by phalloidin-Alexa-647 (blue). Scale bar: 50µm. (0.49 MB PDF) [file ppat.1000711.s003.pdf]

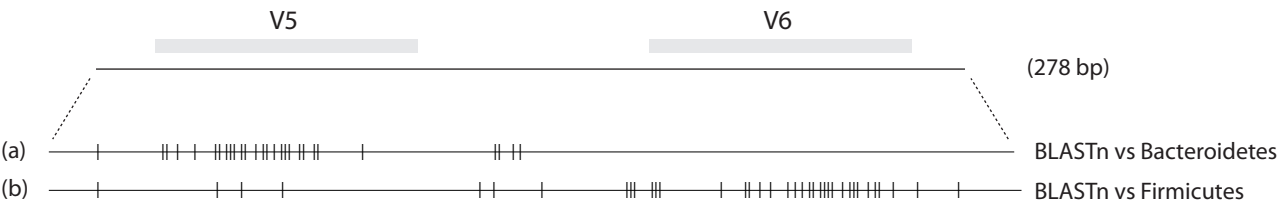

Supplement: Figure S4 — Example for a chimeric sequence read obtained by pyrosequencing. The figure depicts an example for a chimeric V5-V6 read. The mismatches of the chimeric read to the best BLASTn hits (best bit-score) using either a non-redundant (nr) Bacteroidetes database (a) or a nr Firmicutes database (b). Using our chimera detection approach, the V5 region of the depicted read was predicted as Firmicutes and its V6 region as Bacteroidetes. (0.08 MB PDF) [file ppat.1000711.s004.pdf]

Stecher, Chaffron, Käppeli et al. Figure S5

Detection of ASF strains by PCR

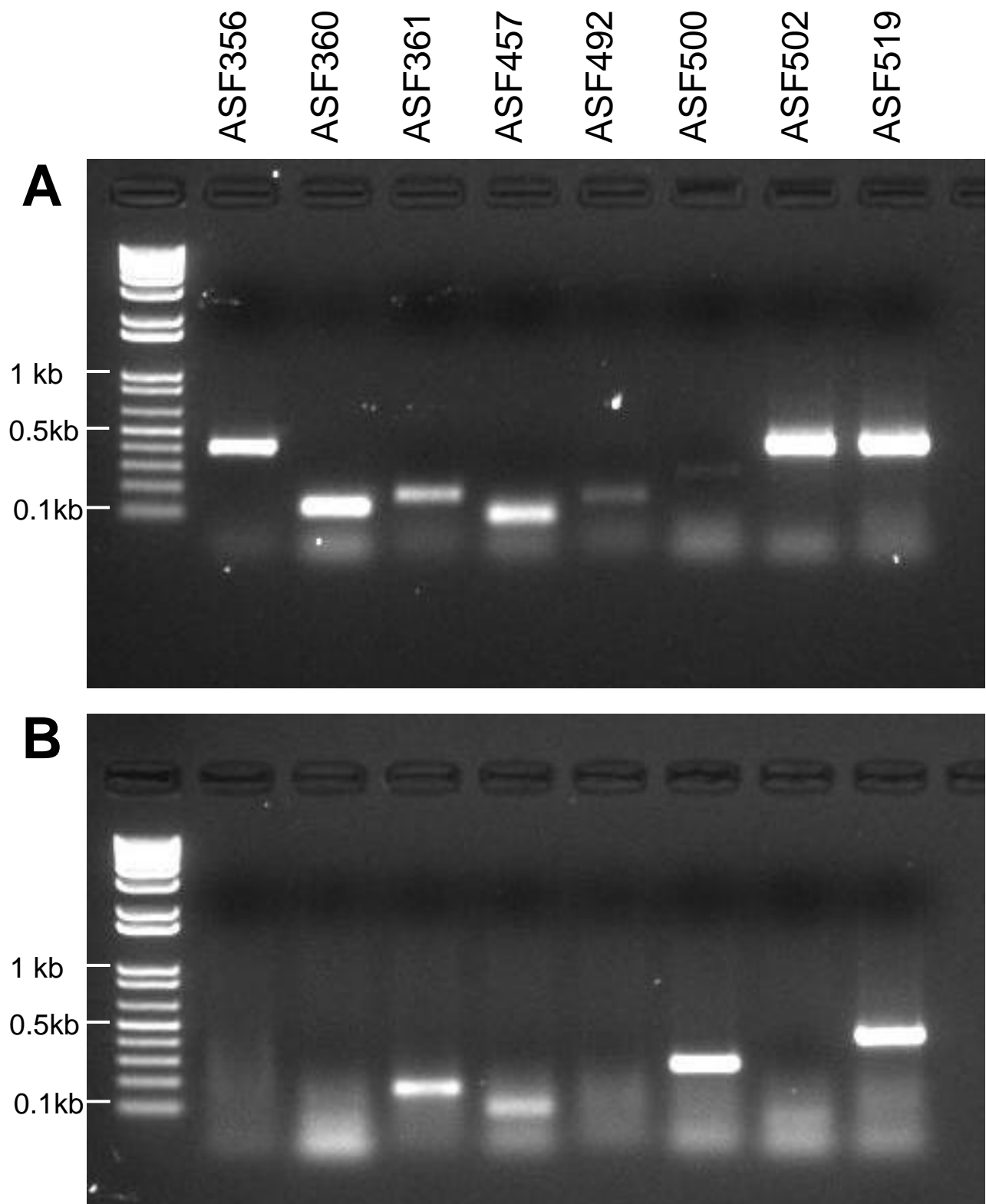

Supplement: Figure S5 — Detection of ASF bacteria in LCM mice by PCR. Fecal bacterial DNA was extracted from LCM mice and used as template for ASF-strain specific PCR. Strain specific PCR for ASF356 (417 bp), ASF360 (131 bp), ASF361 (182 bp), ASF457 (95 bp), ASF492 (167 bp), ASF500 (285 bp), ASF502 (427 bp), and ASF519 (429 bp) (expected sizes shown in parentheses) was performed as described [26]. Linearized plasmids as positive control, containing the 16SrRNA gene of each ASF strain (A) or fecal bacterial DNA was used as template (B). Only strains ASF361, ASF457, ASF500 and ASF519 could be detected in the feces of LCM mice (here only 1 representative PCR result shown). (0.02 MB PDF) [file ppat.1000711.s005.pdf]

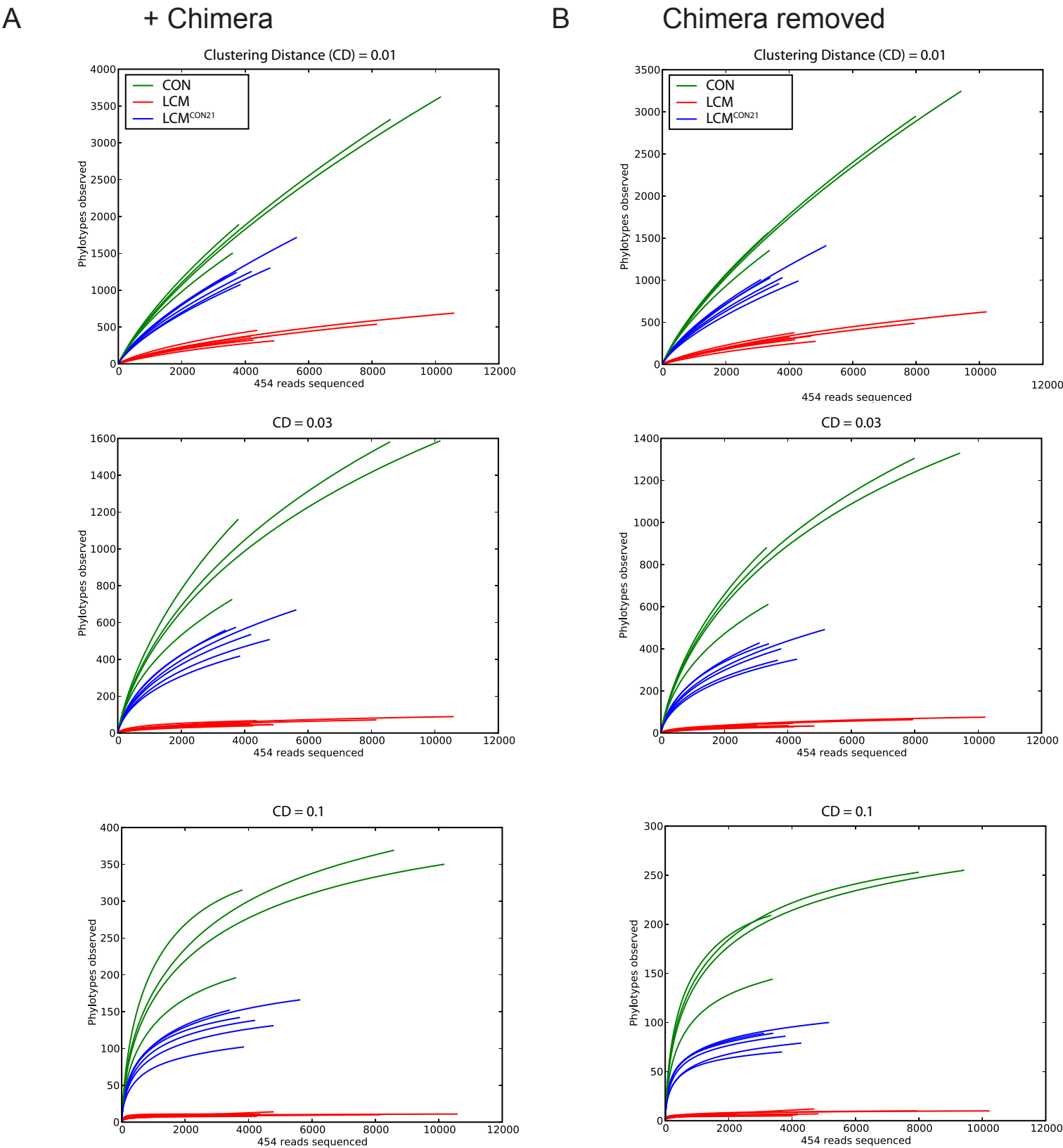

Supplement: Figure S6 — Collectors' curves of LCM, LCMCON21 and CON mice reveal different complexity. Collectors' curves were created for different CD (0.1; 0.03; 0.01) for each mouse from the total number of filtered sequences (A) or from chimera-removed sequences (B). CON mice (green), LCM mice (red) and LCMCON21 mice (blue). (0.68 MB PDF) [file ppat.1000711.s006.pdf]

Taxonomy resolution obtained via reference sequences and via RDP-classifier

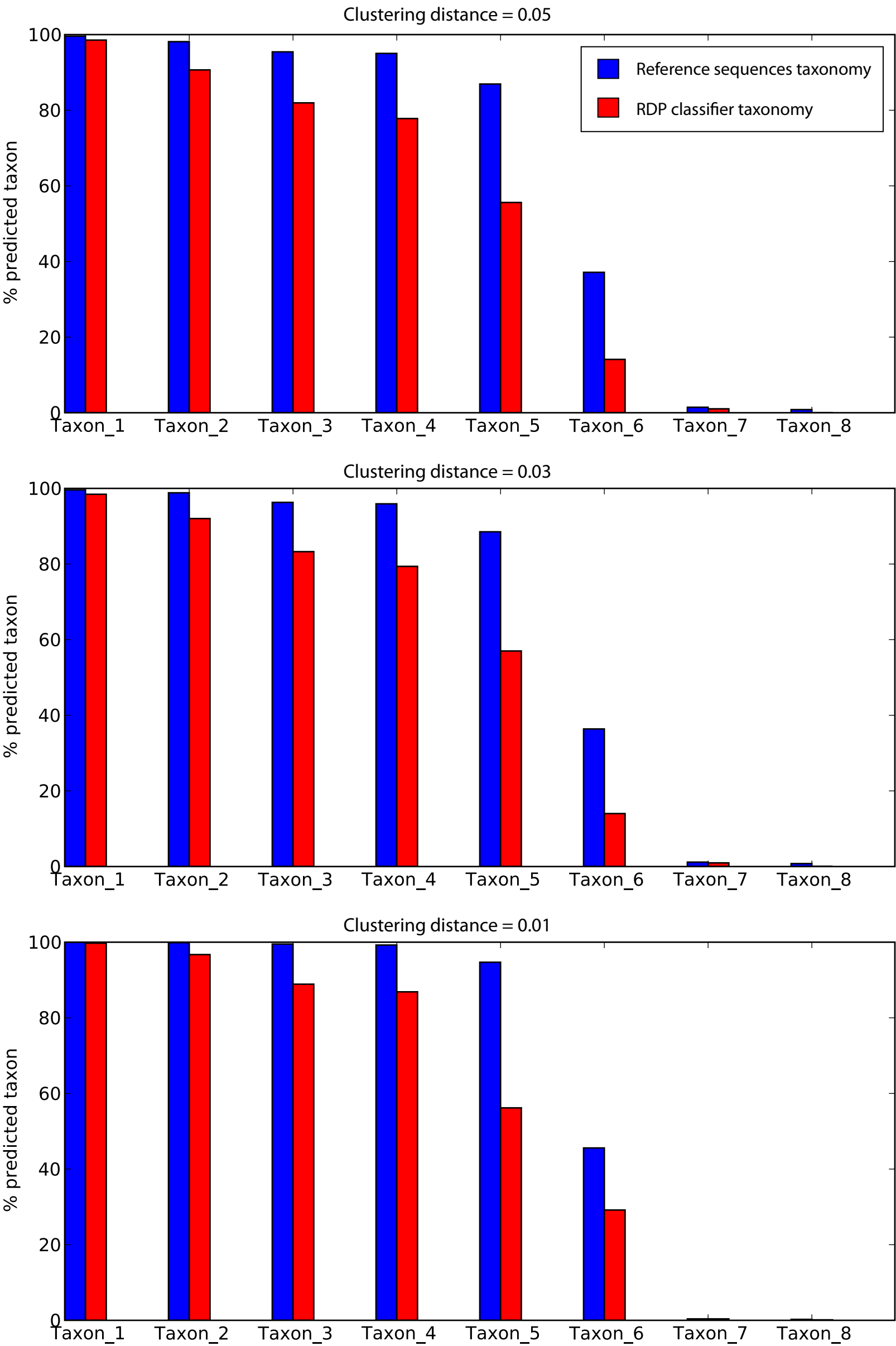

Supplement: Figure S7 — Taxonomy resolution obtained via reference sequences and via RDP-classifier. OTU taxonomy assignment was inferred preferentially using reference sequences annotations (if present in the cluster) or using RDP classifier predictions on the 454 reads. For OTUs containing both reference sequences and reads, we compared the resolution of taxonomies assigned by majority vote via both approaches. For each clustering distance tested and for all taxon levels, a more resolved taxonomy was obtained using the reference sequences annotations. (0.10 MB PDF) [file ppat.1000711.s007.pdf]

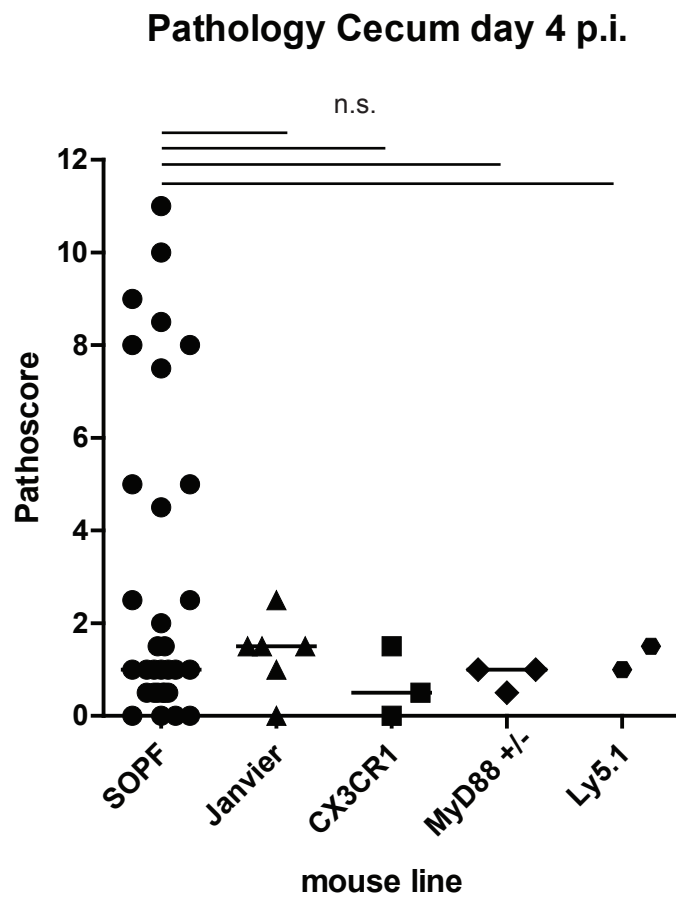

Supplement: Figure S9 — Development of Salmonella-induced gut inflammation in mice with differential fecal E. coli titres. Groups of normal unmanipulated CON mice (6–12 weeks; symbols indicate different sources; x-axis) were infected with 5×10^7^ cfu S. Enteritidis wild type by gavage and sacrificed at day 4 p.i. (see Fig. 5A). Cecal pathology of mice was analyzed in HE-stained tissue sections (see Materials and Methods). (0.14 MB PDF) [file ppat.1000711.s009.pdf]

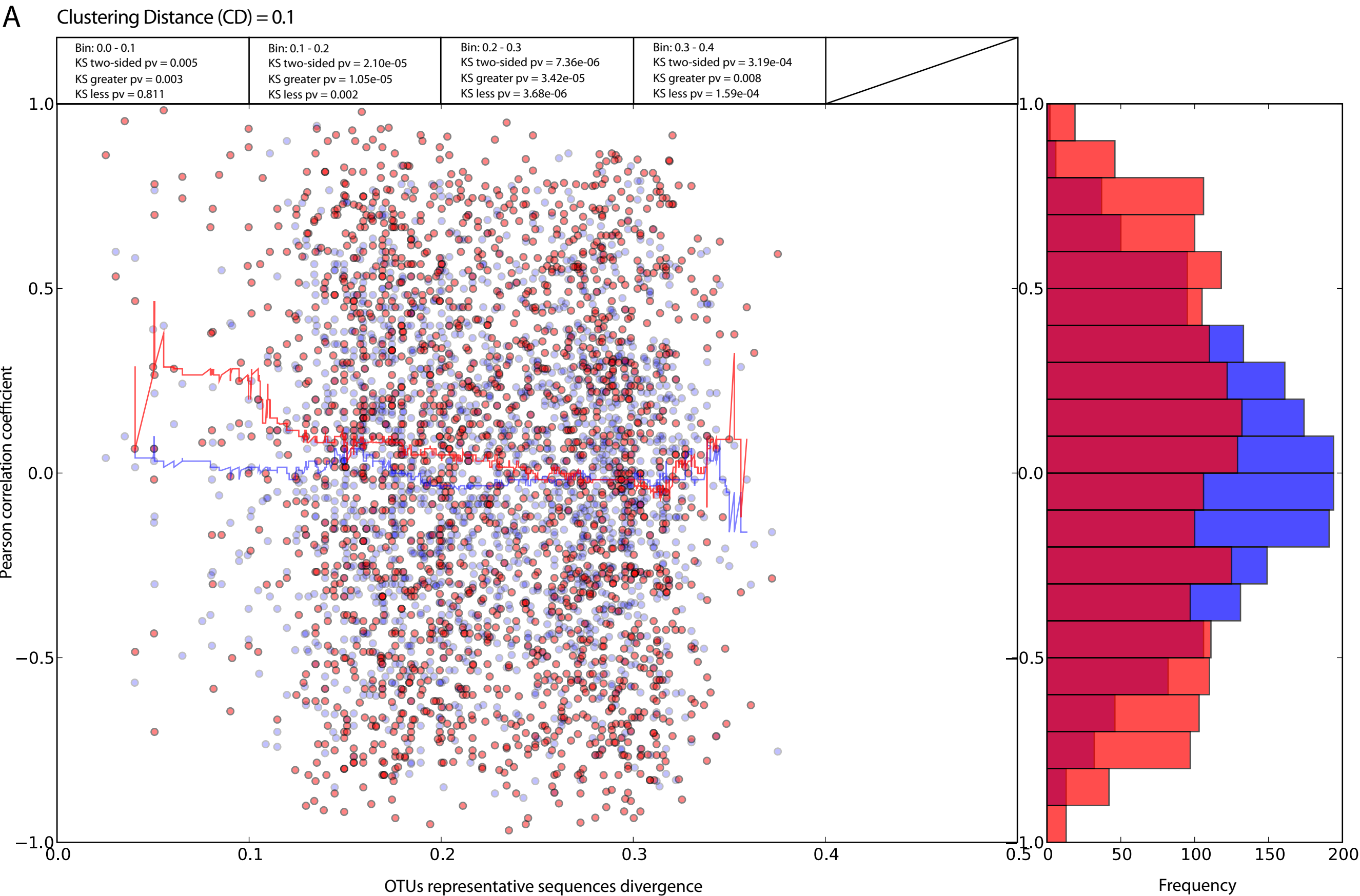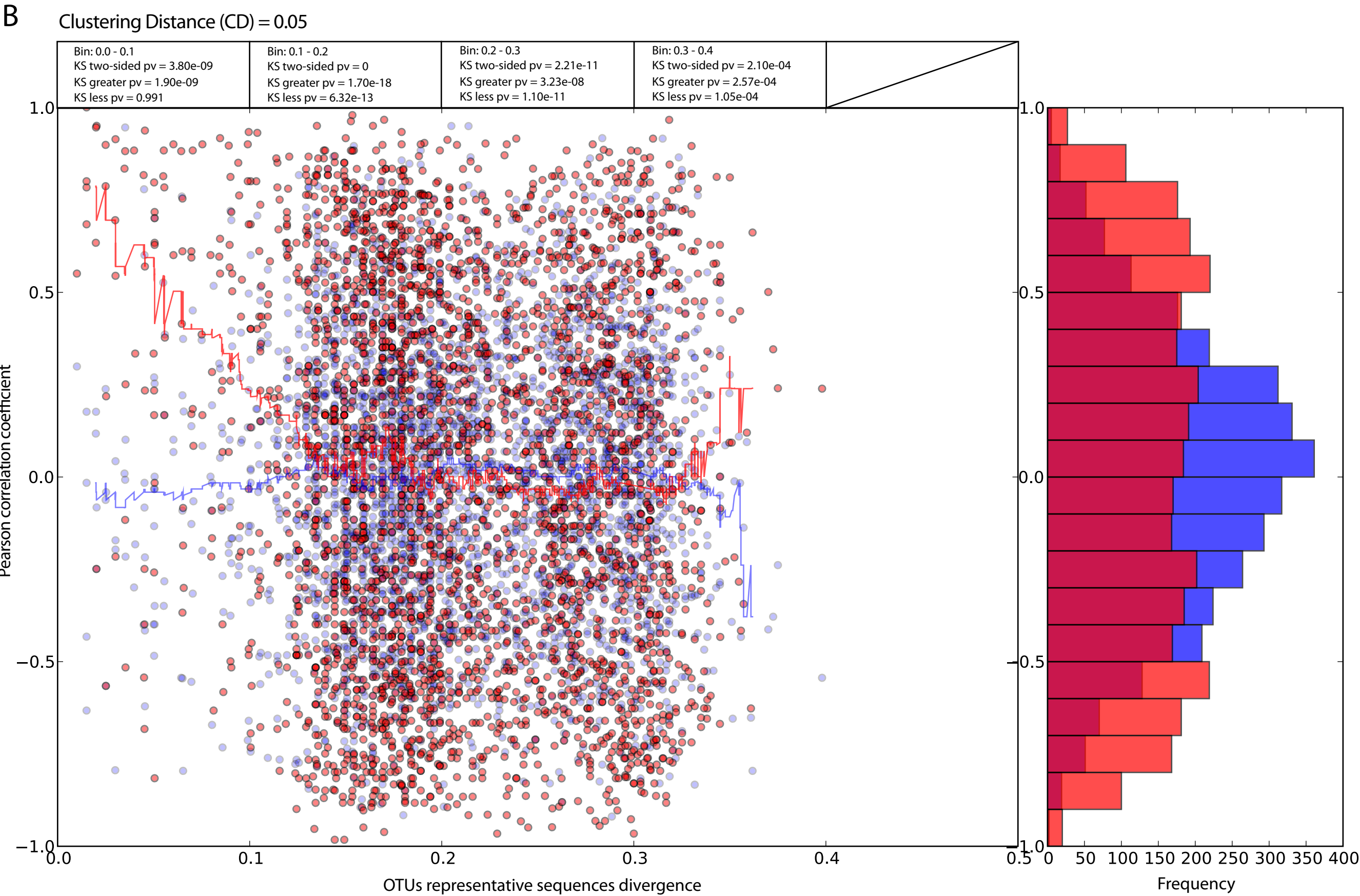

Supplement: Figure S10 — In CON mice, related bacterial lineages are preferentially observed together (quantitative co-occurrence). For all possible pairs of detected OTUs (i.e. present in 2/3 of the analyzed mice; A, CD = 0.1 and B, CD = 0.05; each dot in the graph represents an OTU-pair), abundance correlations (y-axis, Pearson) were computed from abundance measurements in 9 distinct CON mice, and plotted against the molecular divergence between their representative 16S sequences (x-axis). Running medians are represented in the corresponding color. We compared the deviation of the actual data on the y-axis (Pearson correlation) from the distribution of the randomized data using the Kolmogorov-Smirnov test. Both two-sided and one-sided hypotheses (greater and less) were tested for each bin of 0.1 on the x-axis (0.0–0.1; 0.1–0.1; 0.2–0.3; 0.3–0.4). Results are indicated in boxes in the upper part of each graph. Pv = P-value; ‘KS two-sided pv’ indicates whether there is a significant difference between the distribution of the data (red dots) and the distribution of the randomized data (blue dots); ‘KS greater pv’ indicates whether the Pearson correlation coefficient of the data (red dots) is significantly higher compared to the random background in (blue dots) in a given bin. ‘KS less pv’ indicates whether the Pearson correlation coefficient of the data (red dots) is significantly lower compared to the random background in (blue dots) in a given bin. The histogram on the right side of the graph represents the cumulative frequencies of the binned Pearson correlation coefficient data. (6.78 MB PDF) [file ppat.1000711.s010.pdf]
