# Supplementary material for: Like Will to Like: Abundances of Closely Related Species Can Predict Susceptibility to Intestinal Colonization by Pathogenic and Commensal Bacteria
Source: PLoS Pathog. 2010 Jan 8;6(1):e1000711. doi: 10.1371/journal.ppat.1000711 (PMC2796170; doi:10.1371/journal.ppat.1000711)

Stecher, Chaffron, Käppeli et *al.* Figure S8

Qualitative microbiota analysis during re-association of LCM mice

A

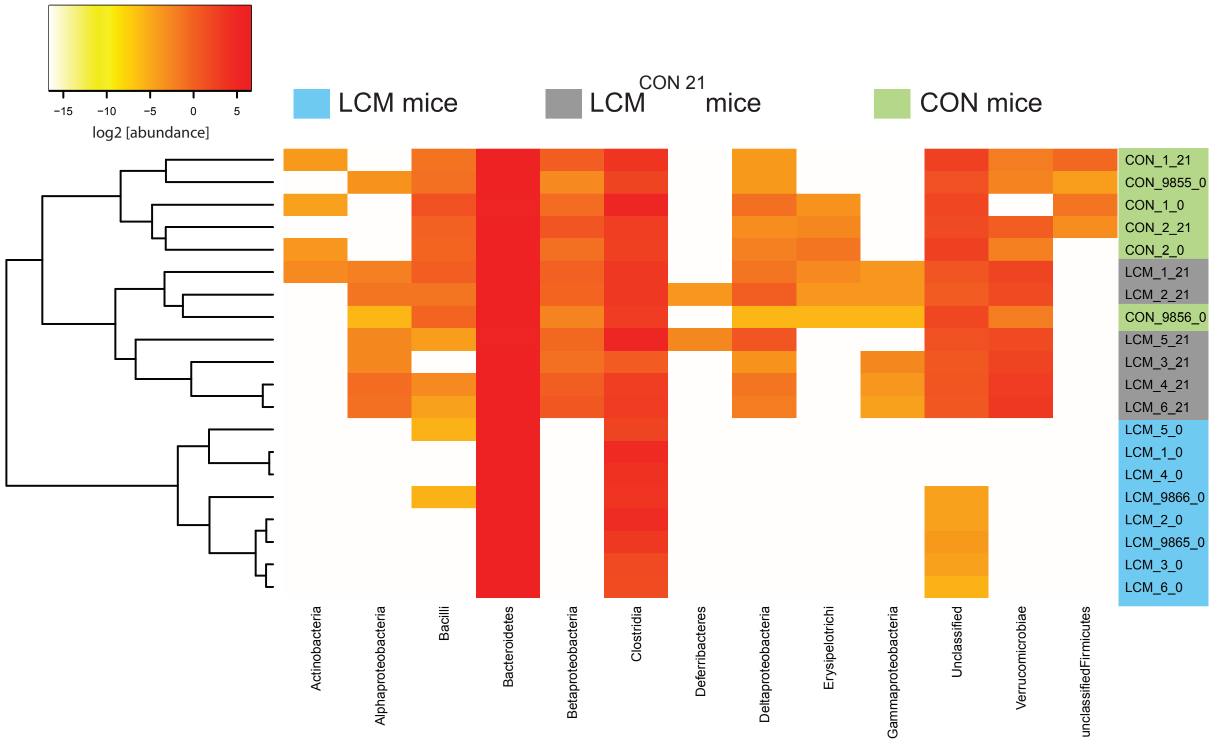

B

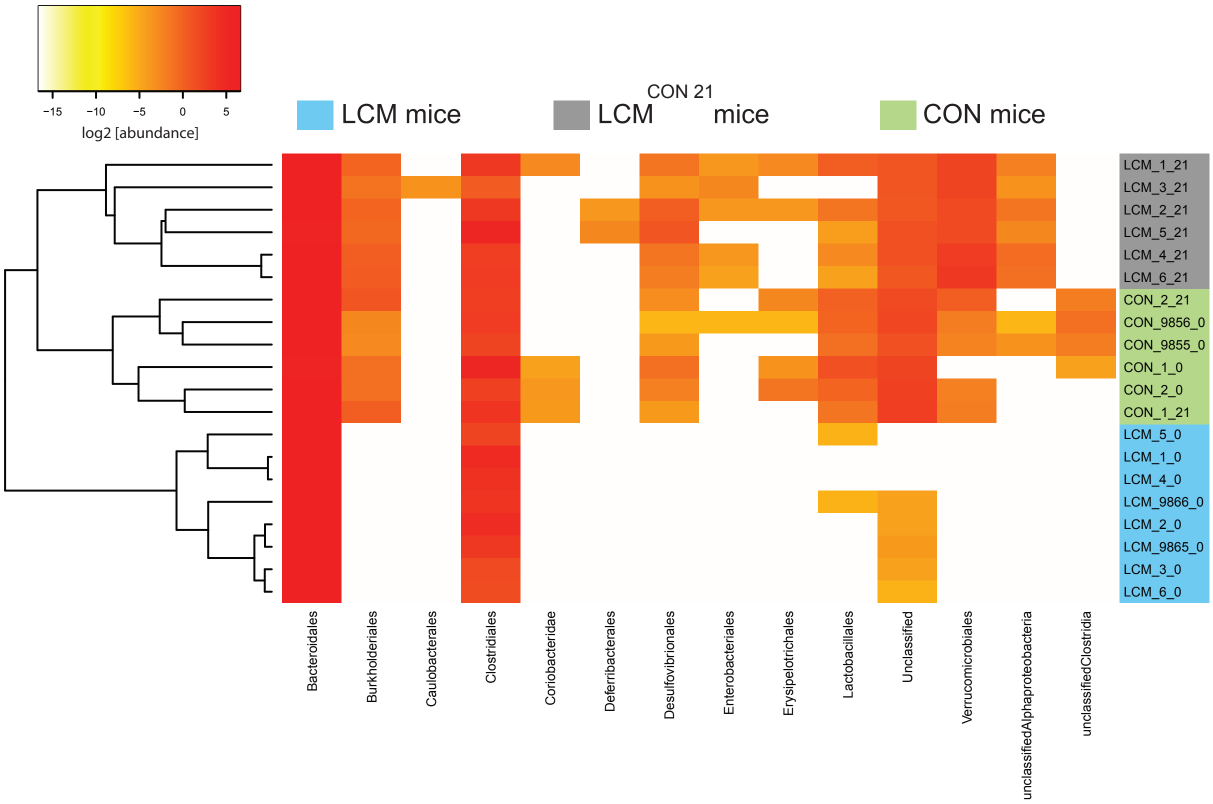

Supplement: Figure S8 — Heatmap showing OTU's distribution in different groups at different phylogenic resolutions. Analysis of fecal microbiota of the mice shown in Fig. 4. Fecal microbiota of unmanipulated LCM mice was analyzed at day 0 (n = 8). 6 of these LCM mice (LCM_1 to LCM_6; blue) were conventionalized in two groups with 2 different CON-donors (CON_1 and CON_2; green) and fecal microbiota analyzed at day 21 (LCM_x_d21; grey). (A) OTUs were sorted according to taxon_2 (class level; X-axis) of (B) according to taxon_3 (order level; X-axix) and average clustering was performed on Euclidean distances calculated between abundance profiles for each mouse and every time-point sampled. Red color indicates high abundance (Log2), yellow color low abundance. CON_9855_d0 and CON_9856_d0 and LCM_9865_d0 and LCM_9866_d0are 2 additional CON or LCM mice, respectively sampled only at day 0. (0.15 MB PDF) [file ppat.1000711.s008.pdf]
