# Supplementary material for: Like Will to Like: Abundances of Closely Related Species Can Predict Susceptibility to Intestinal Colonization by Pathogenic and Commensal Bacteria
Source: PLoS Pathog. 2010 Jan 8;6(1):e1000711. doi: 10.1371/journal.ppat.1000711 (PMC2796170; doi:10.1371/journal.ppat.1000711)
Supplement: Table S1 — Parameters of microbial complexity of CON-donors day 0 (n = 4). (0.06 MB DOC) [file ppat.1000711.s011.doc]

**Table S1. Parameters of microbial complexity of CON-d**onors day 0 (n=4)

|  | **OTU number** | | **Shannon*** | | **Chao1‡** | | **Evenness#** | |
| --- | --- | --- | --- | --- | --- | --- | --- | --- |
| **Distance** | **+ Chi** | **-Chi** | **+ Chi** | **-Chi** | **+ Chi** | **-Chi** | **+ Chi** | **-Chi** |
| **0.01** | **1682.5±596.9** | **1421.5±530.6** | **6.2±0.3** | **5.9±0.3** | **3492.9±1051.4** | **2759±844.2** | **0.83±0.02** | **0.82±0.02** |
| **0.03** | **970.75±290.1** | **767.25±232.9** | **5.4±0.4** | **5.2±0.3** | **1570±402** | **1148±263** | **0.79±0.03** | **0.79±0.03** |
| **0.05** | **662.25±186.2** | **499±138.7** | **4.9±0.4** | **4.7±0.4** | **965±273.8** | **664±175.4** | **0.77±0.04** | **0.76±0.04** |
| **0.1** | **256.5±63.3** | **180±43.8** | **4.0±0.4** | **3.8±0.4** | **284±73.3** | **200±48.5** | **0.73±0.05** | **0.72±0.06** |
| **0.2** | **49±9.8** | **33±7.8** | **2.7±0.3** | **2.4±0.3** | **49±9.7** | **35±9.9** | **0.7±0.07** | **0.68±0.05** |

***** The **Shannon-index** (H') is a measure of species diversity taking into account the number of species and the evenness of the species. i =number of species: p_i_= relative abundance of species i; H' is maximal if all species are present at equal abundance (H^max^ = log(1/i); i = number of species).


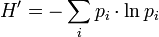


**# Species Evenness**: H'/H^max^; (0-1) An E value of 1 means that the abundance of all species is the same.

**‡** The **Chao1 estimato**r estimates total species richness as


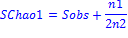


|  |  |
| --- | --- |

where *S*_obs_ is the number of observed species, *n*_1_ is the number of singletons (species captured once), and *n*_2_ is the number of doubletons (species captured twice) [1].

## Supplementary References

1. Hughes JB, Hellmann JJ, Ricketts TH, Bohannan BJ (2001) Counting the uncountable: statistical approaches to estimating microbial diversity. Appl Environ Microbiol 67: 4399-4406.
