# Supplementary material for: Like Will to Like: Abundances of Closely Related Species Can Predict Susceptibility to Intestinal Colonization by Pathogenic and Commensal Bacteria
Source: PLoS Pathog. 2010 Jan 8;6(1):e1000711. doi: 10.1371/journal.ppat.1000711 (PMC2796170; doi:10.1371/journal.ppat.1000711)
Supplement: Table S2 — Parameters of microbial complexity of LCM-recipients day 0 (n = 8). (0.03 MB DOC) [file ppat.1000711.s012.doc]

**Table S2. Parameters of microbial complexity of LCM-recipients day 0 (n=8**)

|  | **OTU number** | | **Shannon*** | | **Chao1‡** | | **Evenness#** | |
| --- | --- | --- | --- | --- | --- | --- | --- | --- |
| **Distance** | **+ Chi** | **-Chi** | **+ Chi** | **-Chi** | **+ Chi** | **-Chi** | **+ Chi** | **-Chi** |
| **0.01** | **154.8±51.5** | **130.9±49.2** | **1.4±0.47** | **1.2±0.42** | **185.5±65.9** | **153±61.5** | **0.28±0.09** | **0.26±0.09** |
| **0.03** | **38.3±9.6** | **28.6±10** | **0.67±0.26** | **0.5±0.22** | **55±13.2** | **41.9±10.7** | **0.18±0.07** | **0.15±0.06** |
| **0.05** | **22.5±4.6** | **16.9±4.9** | **0.58±0.25** | **0.43±0.21** | **28.3±7** | **20.8±5.8** | **0.18±0.08** | **0.15±0.07** |
| **0.1** | **8.9±1.7** | **6.8±2.3** | **-** | **-** | **-** |  | **0.24±0.1** | **0.22±0.14** |
| **0.2** | **-** | **-** |  | **-** | **-** |  |  |  |
