# Supplementary material for: Like Will to Like: Abundances of Closely Related Species Can Predict Susceptibility to Intestinal Colonization by Pathogenic and Commensal Bacteria
Source: PLoS Pathog. 2010 Jan 8;6(1):e1000711. doi: 10.1371/journal.ppat.1000711 (PMC2796170; doi:10.1371/journal.ppat.1000711)
Supplement: Table S3 — Parameters of microbial complexity of LCM-recipients day 21 (n = 6). (0.03 MB DOC) [file ppat.1000711.s013.doc]

**Table S3. Parameters of microbial complexity of LCM-recipients day 21 (n=6**)

|  | **OTU number** | | **Shannon*** | | **Chao1‡** | | **Evenness#** | |
| --- | --- | --- | --- | --- | --- | --- | --- | --- |
| **Distance** | **+ Chi** | **-Chi** | **+ Chi** | **-Chi** | **+ Chi** | **-Chi** | **+ Chi** | **-Chi** |
| **0.01** | **756.5±115.2** | **589.3±81.01** | **5.1±0.2** | **4.8±0.2** | **1371.1±260.6** | **1014±185.1** | **0.77±0.03** | **0.75±0.03** |
| **0.03** | **409.2±59.6** | **295.3±33.9** | **4.3±0.3** | **4.0±0.2** | **622.2±102** | **429.4±60.6** | **0.71±0.03** | **0.7±0.03** |
| **0.05** | **279±44.9** | **188.3±23.2** | **3.9±0.3** | **3.6±0.3** | **392.4±78.7** | **254.7±51.7** | **0.7±0.04** | **0.7±0.04** |
| **0.1** | **118.2±18.1** | **73.2±9.6** | **3.3±0.2** | **3.0±0.2** | **129±20.6** | **84.2±16.6** | **0.69±0.03** | **0.7±0.03** |
| **0.2** | **27.6±2.3** | **19.4±1.2** | **2.4±0.2** | **2.1±0.2** | **27.7±2.3** | **19.6±1.3±** | **0.72±0.04** | **0.72±0.05** |
